# Smartphone App Using Mindfulness Meditation for Women With Chronic Pelvic Pain (MEMPHIS): Protocol for a Randomized Feasibility Trial

**DOI:** 10.2196/resprot.7720

**Published:** 2018-01-15

**Authors:** Elizabeth Ball, Sian Newton, Brennan C Kahan, Gordon Forbes, Neil Wright, Clara Cantalapiedra Calvete, Harry A L Gibson, Ewelina Rogozinska, Carol Rivas, Stephanie J C Taylor, Judy Birch, Julie Dodds

**Affiliations:** ^1^ Department of Obstetrics and Gynaecology Barts Health NHS Trust London United Kingdom; ^2^ Women’s Health Research Unit Barts and the London School of Medicine and Dentistry Queen Mary University of London London United Kingdom; ^3^ Centre for Primary Care and Population Health Queen Mary University of London London United Kingdom; ^4^ Pragmatic Clinical Trials Unit Queen Mary University of London London United Kingdom; ^5^ Clinical Trial Service Unit and Epidemiological Studies Unit University of Oxford Oxford United Kingdom; ^6^ Social Science Research Unit University College London London United Kingdom; ^7^ Pelvic Pain Support Network Poole United Kingdom

**Keywords:** randomized controlled trial, pelvic pain, chronic pain, mobile applications, mindfulness, meditation

## Abstract

**Background:**

Female chronic pelvic pain (CPP) is defined as intermittent or constant pelvic or lower abdominal pain occurring in a woman for at least 6 months. Up to a quarter of women are estimated to be affected by CPP worldwide and it is responsible for one fifth of specialist gynecological referrals in the United Kingdom. Psychological interventions are commonly utilized. As waiting times and funding capacity impede access to face-to-face consultations, supported self-management (SSM) has emerged as a viable alternative. Mindfulness meditation is a potentially valuable SSM tool, and in the era of mobile technology, this can be delivered to the individual user via a smartphone app.

**Objective:**

To assess the feasibility of conducting a trial of a mindfulness meditation intervention delivered by a mobile phone app for patients with CPP. The main feasibility objectives were to assess patient recruitment and app adherence, to obtain information to be used in the sample size estimate of a future trial, and to receive feedback on usability of the app.

**Methods:**

Mindfulness Meditation for Women With Chronic Pelvic Pain (MEMPHIS) is a three-arm feasibility trial, that took place in two hospitals in the United Kingdom. Eligible participants were randomized in a 1:1:1 ratio to one of three treatment arms: (1) the intervention arm, consisting of a guided, spoken mindfulness meditation app; (2) an active control arm, consisting of a progressive muscle relaxation app; and (3) usual care (no app). Participants were followed-up for 6 months. Key feasibility outcomes included the time taken to recruit all patients for the study, adherence, and estimates to be used in the sample size calculation for a subsequent full-scale trial. Upon completion of the feasibility trial we will conduct focus groups to explore app usability and reasons for noncompliance.

**Results:**

Recruitment for MEMPHIS took place between May 2016 and September 2016. The study was closed March 2017 and the report was submitted to the NIHR on October 26, 2017.

**Conclusions:**

This feasibility trial will inform the design of a large multicentered trial to assess the clinical effectiveness of mindfulness meditation delivered via a smartphone app for the treatment of CPP.

**Trial Registration:**

ClinicalTrials.gov: NCT02721108; https://clinicaltrials.gov/ct2/show/NCT02721108 (Archived by WebCite at http://www.webcitation.org/6wLMAkuaU); BioMed Central: ISRCTN10925965; https://www.isrctn.com/ISRCTN10925965 (Archived by WebCite at http://www.webcitation.org/6wLMVLuys)

## Introduction

Female chronic pelvic pain (CPP) is defined as intermittent or constant pain in the lower abdomen or pelvis of a woman for at least 6 months, which is not exclusively associated with menstruation or intercourse, and not associated with pregnancy [1]. CPP affects up to 24% of women worldwide [2], accounts for 20% of gynecological clinic referrals in the United Kingdom [3], and has a considerable impact on patients’ quality of life and their income. There are no recent estimates of the cost of CPP, but endometriosis-associated pain alone costs the United Kingdom economy £8.2 billion per year in treatment, loss of work, and health care costs [4], so the cost of general CPP is likely to be even higher. Despite costly interventions, CPP is often resistant to surgical and medical treatment. Both psychosocial causes (such as a history of sexual abuse) and somatic causes (eg, endometriosis, pelvic inflammatory disease, bladder pain syndrome) can contribute to CPP [1]. High levels of depression and anxiety are commonly associated with CPP, but are often not addressed in this population [5].

These multifactorial causes require a multidimensional approach, which is not routinely offered in gynecology clinics [6]. Evidence from randomized controlled trials (RCTs) suggests that a holistic approach using psychological interventions may be superior to primary surgery [7]. Although psychological treatment is provided across the National Health Service (NHS), mostly in the context of the primary care program *Improving Access to Psychological Therapies*, there are problems with capacity, waiting times, and the overall number of patients that are able to access services. Alternatively, supported self-management (SSM) is now recognized as a tool that empowers patients to better cope with their condition [8].

Mindfulness-based therapy is currently creating lively research interest. Two recent systematic reviews report positive effects on somatization disorders [9] and psychological stress [10]. A further systematic review carried out by our research team [11], which examined 15 RCTs for online mindfulness meditation, found small but significant beneficial impacts on depression, anxiety, well-being, and mindfulness, and a moderate effect on stress, with guided programs proving more effective than unguided ones. We are only aware of one ongoing randomized Danish study of mindfulness in patients with endometriosis-specific CPP (NCT02761382).

Our review also found that mindfulness meditation to treat CPP had a promising effect on patient well-being [11], as demonstrated in pilot studies on CPP and larger RCTs on other types of chronic pain. We are therefore investigating the feasibility of a full RCT for mindfulness in CPP, as mindfulness has great potential as a self-management tool that could be used as part of a holistic approach to CPP.

More convenient delivery methods have been called for as an alternative to the 8-week face-to-face sessions required for the standard mindfulness based stress reduction (MBSR) courses [12]. During a patient and public involvement (PPI) session we held to help design our study, CPP patients expressed a preference for receiving the intervention through a smartphone app, as it is portable and could be accessed when and where they liked.

The systematic review showed that mindfulness meditation helps chronic pain patients accept pain better and helps to alleviate anxiety and depression [11], which are particularly common in this population [5]. One of the suggested mechanisms of mindfulness meditation is the uncoupling of sensory aspects from the evaluative and emotional aspects of pain through mindful awareness and meditation. Unlike cognitive behavioral therapy, which is goal oriented, mindfulness meditation relies on nonjudgmental observation. By distancing themselves from painful sensations and thoughts, instead of being alarmed, patients can achieve greater acceptance of chronic pain rather than permanently wanting to control it.

### Systematic Review

Our systematic search and review of the literature on mindfulness meditation in CPP (July 2013; updated May 2017) was designed to investigate prior research in the area before commencing our study. Our systematic review was conducted in line with current standards [13]. We searched MEDLINE (via OVID), EMBASE, PsychINFO, and AMED without language restrictions from database inception to July 2013, and subsequently updated the search in May 2017. The databases were searched for relevant studies using the following key words and word variants: *chronic pain* or *pelvic pain*, and *meditation* or *mindfulness* or *Vipanassa* or *mindfulness based stress reduction* or *mindfulness based intervention* or *mindfulness based therapy*. The reference lists from the obtained articles were examined for additional articles. We also hand-searched all relevant systematic reviews and, if necessary, we approached the authors for missing relevant information.

The first search identified two small, nonrandomized pilot trials investigating the effect of mindfulness meditation on pelvic pain (n=22) [14], and endometriosis (n=10) in particular [15]. Both small studies were uncontrolled. In the study on CPP, significantly improved scores were reported for daily maximum pain, physical function, mental health (*P*=.01), and social function [14]. The mindfulness scores improved significantly in all measures [14]. In endometriosis patients, significant improvements were reported for bodily pain, general health, and vitality [15].

Since that time, two more studies have been published. Kanter et al investigated the effect of mindfulness compared to usual care in an RCT of patients with bladder pain syndrome (n=20) [16]. Outcome measures relating to empowerment and self-management improved significantly [16]. A small pilot study on military women with CPP (n=15) showed a nonsignificant reduction in pain and increase in mindfulness measures [12]. The authors of this study called for simpler formats of teaching mindfulness than the 8-week standard MBSR, which four studies used [12].

Given this paucity of data on mindfulness in CPP, we expanded our systematic review of mindfulness meditation to include its use in other chronic pain conditions (back pain, headaches, fibromyalgia, and diabetic neuropathy), as we assumed that any benefits in these conditions might also be seen in CPP. Previous systematic reviews of these conditions had a number of limitations, such as not reporting effect sizes [17-19].

We identified 534 relevant citations, and 9 RCTs [20-28] were included in the review [11]. Most studies were of moderate quality, but sample sizes were generally small (from 65 to 259 women). Our results showed mindfulness-based meditation reduced depression levels in chronic pain patients (standardized mean difference [SMD] -0.31; 95% CI -0.52 to -0.10; I^2^=0%) and anxiety (SMD -0.21; 95% CI -0.45 to 0.03; I^2^=0%). Pain acceptance was also improved (SMD 0.34; 95% CI 0.09-0.59). No significant changes were seen in quality of life, anxiety, pain scores, or the emotional response to pain.

There are few published robust trials of apps to assist better self-management of chronic conditions. A Cochrane review of apps for asthma found only two studies and concluded there was insufficient evidence to advise patients on their usefulness [29]. Although CPP is as common as back pain and asthma [30], there are no RCTs that are investigating mindfulness meditation in CPP. Mindfulness meditation had shown a promising effect on patient well-being in uncontrolled pilot studies on CPP and larger RCTs on other types of chronic pain [11]. Given the high levels of depression and anxiety in CPP patients, combined with difficult access to psychological treatments, this approach could address a gap both in knowledge and patient care.

### Development of the Mindfulness Meditation App Module

Headspace [31], a company that had already developed and successfully established a mindfulness meditation app, was approached to develop a module for meditation for chronic pain. This module was incorporated into the existing library of app content. The pain module can be accessed after participants have completed the 10-day foundation program.

### Aims and Objectives

The overall aim of this study is to assess the feasibility of implementing a full scale, multicenter RCT to test the efficacy of a mindfulness meditation intervention delivered by a mobile phone app for patients with CPP. The primary objectives are: (1) to provide feasibility data for a large multicenter RCT aimed at rigorously testing mindfulness meditation in patients with CPP (the full-scale trial will assess the effectiveness of the mindfulness meditation app in patients with CPP in a national multicenter RCT); and (2) to determine whether this app can be seamlessly integrated into clinical practice, especially CPP pathways.

## Methods

### Design

Mindfulness Meditation for Women With Chronic Pelvic Pain (MEMPHIS) is a three-arm randomized feasibility trial. Approval was received by Camden and Kings Cross Ethics Committee in February 2016 (15/LO/1967).

#### Inclusion Criteria

To be eligible for the MEMPHIS study, women were required to meet the following criteria: (1) aged 18 years or over; (2) have organic and nonorganic CPP lasting for 6 months or more; (3) be capable of understanding the information provided, and be able to understand simple English as is used in the app; and (4) give written informed consent.

#### Exclusion Criteria

Patients who met the following criteria were ineligible to participate: (1) no access to a personal computer or smartphone, or (2) current users of the Headspace app content available to the public.

#### Study Design

**Figure 1:**
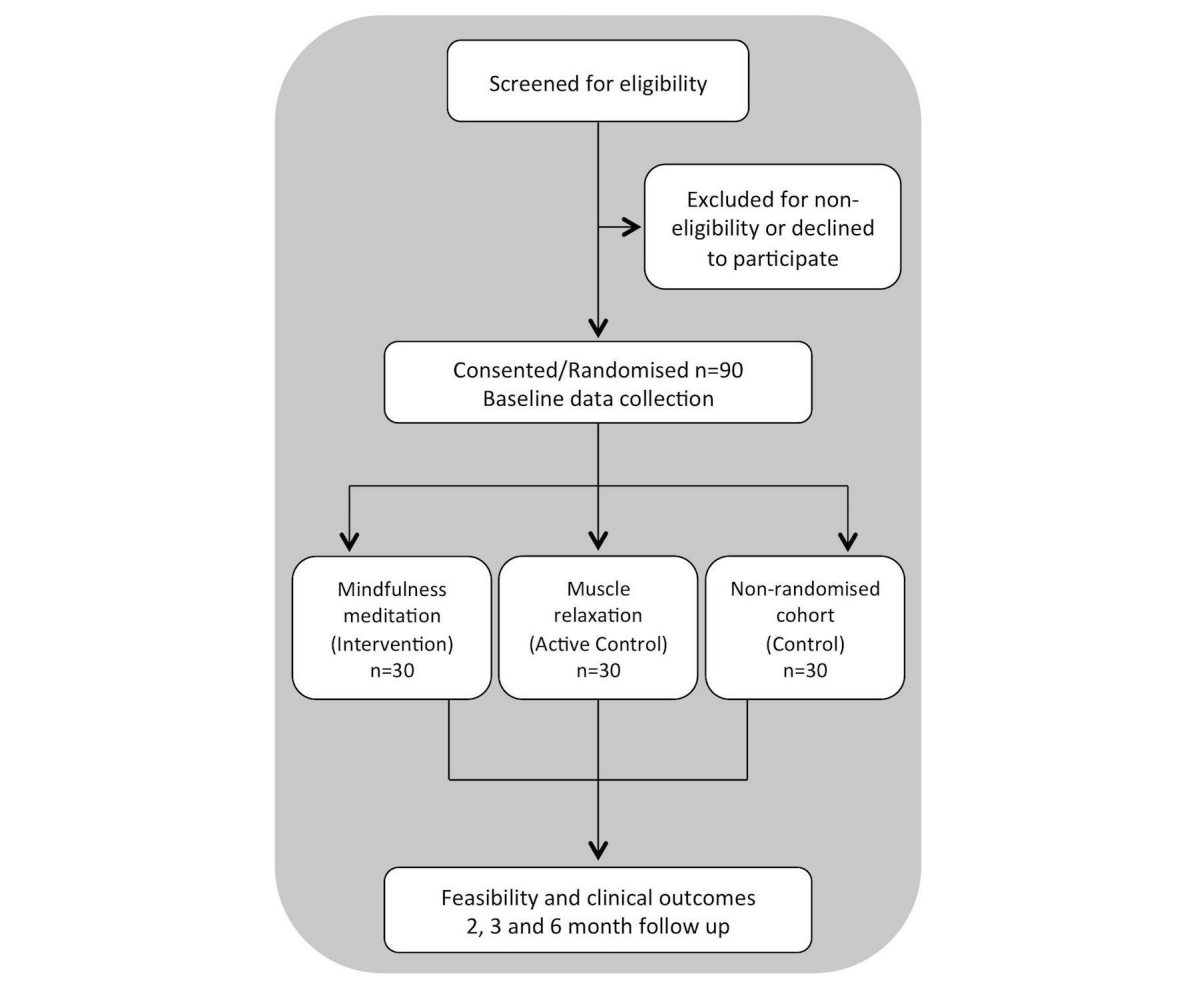
Participant flow showing eligibility, enrolment, randomization and follow-up.

MEMPHIS is a three-arm randomized feasibility trial. All eligible women referred to the CPP clinics at the Royal London and Whipps Cross Hospitals (both new and existing patients) were approached to take part in the study. The setting of the study was NHS Tertiary care hospitals. After informed consent, we randomized eligible women in a 1:1:1 ratio (30 participants in each group) to one of the three treatment groups: (1) *Intervention*, consisting of 60 days of the app delivering mindfulness meditation content (in addition to usual care); (2) *Active control*, consisting of 60 days of the app delivering progressive muscle relaxation content (in addition to usual care); and (3) *Treatment as usual* consisting of usual care. See Figure 1 for a flow chart of the study.

### Outcomes

#### Feasibility Outcomes Collected From Participants

Several parameters were collected regarding the participants (Textbox 1).

#### Participant Focus Groups

Usability and integration into clinical practice were explored in two postintervention focus groups at each recruiting site: one for each app group. All participants allocated to each app group were invited to a session to take place in a private room at or near the clinic they were recruited from. We aimed to recruit approximately 15 app participants from the study participants. We offered a telephone interview as an alternative to any participant who was unable to attend their focus group.

Discussions were structured around the app System Usability Scale (SUS) and additional questionnaire, and were expected to take two hours to complete. Discussions were recorded and literal themes on integration and usability will be evaluated for in-depth information. This information will be considered, as well as adherence to the app, as an indirect measure of acceptability. We will determine primary and secondary outcomes of interest, from the perspective of patients, for a full-scale trial. This aim will involve asking participants who were randomized to the app groups to discuss and prioritize outcomes. Obstacles to recruitment will also be explored.

#### Health Care Practitioner Focus Groups

The health care practitioners that were involved with the trial were invited to attend focus groups. A purpose-made topic guide was used to structure focus groups with service providers, which were held in private rooms at the university or clinic, and based on the Normalization Process Theory toolkit [34] and Diffusion of Innovations Theory [35]. Topics were used as a prompt by the facilitator to identify any emergent or residual problems that might act as a barrier to use and effectiveness of the app in the RCT, and implementation into practice.

The service providers were asked to consider their role and their organization, to suggest and discuss any issues related to integration, and to also suggest potential solutions (unlike conventional qualitative research focus groups). Discussions considered relative advantage versus existing practices, compatibility with existing practices, simplicity and ease of integration, trialability and reinvention of the process, feedback (eg, can clinicians see that patients benefit?), and peer-to-peer networking. We will use our findings to develop our integration approach to be further explored in the subsequent full trial. Obstacles to recruitment will also be explored.

#### Clinical Outcomes

Clinical outcomes measures have been selected based on the findings of our systematic review [11] and for their relevance to women with CPP. All scales are validated. We will use the clinical outcomes listed in Textbox 2.

All clinical outcomes were self-reports completed by study participants at baseline, 60 days, 3 months, and 6 months postrandomization (Multimedia Appendix 2).

**Feasibility outcomes collected from patients**

- Time taken to recruit all patients for the study (measured from the day recruitment opens until the day the 90th patient was randomized).
- Estimates to be used for the sample size calculation of the full-scale randmoized control trial:
  - The estimated standard deviation for pain acceptance (as measured by the Chronic Pain Acceptance Questionnaire [CPAQ-8] [32])
  - The dropout rate defined by the proportion of participants who never return or answer a follow-up questionnaire at 6 months postrandomization
  - The proportion of participants who do not return a follow-up questionnaire, but do answer the questionnaire by phone at 6 months postrandomization
- Patient adherence to app use will be investigated using the following outcomes:
  - Number of days (within the first 60 days from randomization) a patient has used the app. The definition of app use was originally defined as having completed 50% of a session. This definition was changed to 90% of a session during the trial (see Multimedia Appendix 1).
  - Whether the patient has used the app on 22 or more days within the first 60 days following randomization
  - Number of weeks (within the first 8 weeks from randomization) during which a patient has used the app on three or more days
  - Whether the patient has used the app on three or more days in 6 or more weeks (within the first 8 weeks from randomization)
  - Whether the patient has used the app on 22 or more days within the first 60 days from randomization, and used the app on three or more days in 6 or more weeks within the first 8 weeks from randomization
- Usability of the app will be measured by:
  - The System Usability Scale [33]
  - A purpose-made nonvalidated questionnaire (Multimedia Appendix 2) developed from patient and public involvement discussion and exploring the issues this group suggested women with chronic pelvic pain might have when using the particular app chosen. For example, we consider its modular format as well as the fit within daily activities, as we had been told from public and patient engagement work that this might be a problem for working women.

**Clinical outcomes**

- Pain acceptance score (as measured by the Chronic Pain Acceptance Questionnaire [CPAQ-8] [32])
- Quality of life score, Physical Functioning subscale (as measured by the RAND 36-item Short Form health survey [SF-36] [36])
- Quality of life score, Social Functioning subscale (as measured by the RAND SF-36)
- Quality of life score, Pain subscale (as measured by the RAND SF-36)
- Quality of life score, General Health subscale (as measured by the RAND SF-36)
- Depression score (as measured by the Hospital Anxiety and Depression Scale [HADS] [37])
- Anxiety score (as measured by the HADS)
- Mindfulness score (as measured by the Cognitive and Mindfulness Scale - Revised [CAMS-R] [38])
- Pain related disability score (as measured by the Chronic Pain Grade [CPG] disability subscale [39])
- Self-efficacy score (as measured by the Pain Self-Efficacy Questionnaire [PSEQ] [40])
- Sexual Health Outcomes score (as measured by Sexual Health Outcomes in Women Questionnaire [SHOW-Q] [41])
- Subjective outcome score (as measured by Measure Yourself Medical Outcome Profile [MYMOP] [42])

### Recruitment

#### Informed Consent Procedures

Women were made aware of the study by health care professionals and through promotional materials. Potentially eligible patients received the Patient Information Sheet (PIS) and were given adequate time (at least 24 hours) to consider the trial. Health care professionals and researchers screened eligibility at the time of outpatient clinic visits before offering participation. Women not interested or not eligible were recorded in a screening log. Eligible patients who were seen in clinics other than pelvic pain and endometriosis clinics were given the PIS and contact details for the researcher so they could benefit from participating in MEMPHIS, if they so wished.

#### Randomization Procedures

After providing informed consent, patients were randomized (maintaining full allocation concealment) in a 1:1:1 ratio to one of the three treatment groups, using permuted blocks without stratification. Block sizes were 27, 30, and 33. Randomization was performed using a centralized Internet service, hosted by the Pragmatic Clinical Trials Unit at Queen Mary University of London.

#### Blinding Following Allocation to Study Arm

The participant and recruiting staff were aware of allocation to either the *Treatment as usual* group or one of the app groups. The participant and recruiting staff were, however, blinded to allocation to the *Intervention* or *Active Control* app groups.

To preserve blinding of participants within the two app arms of the study, each group used the same app and hearing instructions for the same duration, delivered by the same narrator. Only the content of the instructions differed. In addition, the PIS and consent forms did not explicitly refer to “mindfulness meditation” or “progressive muscle relaxation.”

Data on whether any unblinding occured with recruiting staff, or if participants were aware of their allocation, were collected immediately after randomization and after 6 months, respectively. Statisticians will be blinded to individual treatment allocations until required for the final analysis.

### Interventions

The researchers encouraged participants to aim to use the app they were randomized to daily, for as many sessions as they felt comfortable with.

#### The Active Intervention: Mindfulness Meditation App

The meditation content was delivered via a structured and progressive course, layering in new techniques and concepts over successive sessions. The course was created and narrated by a former monk, Andy Puddicombe, drawing on a secularized version of the techniques he was taught over 10 years in monasteries around the world.

The techniques used in the *Intervention* are shown in Table 1. The first 30 days covered basic techniques, assuming no previous experience of meditation. The second 30 days focused specifically on the use of these techniques with respect to pain. The duration of individual sessions built over time. Days 1-10 involved 10 minutes/day, days 11-20 involved 15 minutes/day, and days 21-60 involved 20 minutes/day. Headspace collected data on adherence to the active intervention.

#### The Active Control App and the Treatment As Usual Group

The Active Control group used the same app, but the app was configured so that they heard a series of nonmeditative progressive muscle relaxation instructions, also narrated by Andy Puddicombe. These sessions were identical every day, except that their duration increased to mirror the increasing duration of the meditation content being listened to by the Intervention group.

In this way, both Intervention and Active Control groups used the same app, and hearing instructions for the same duration, delivered by the same narrator. Only the content of the instructions differed. Headspace collected data on adherence to the active control.

The control group consisting of treatment as usual (watch and wait, medication, and/or surgery) was included to investigate if any app intervention makes a difference to well-being and to ascertain dropout rates for the full-scale trial in patients who perceive that they are receiving no intervention.

### Schedule of Assessment

Health outcome measures were collected at baseline and immediately after the intervention at 60 days, 3 months, and 6 months. The time points were chosen to obtain information close to finishing the intervention to assess immediate effects, at a mid-point, and after 6 months to assess longer-term effects. App satisfaction/usability measures were collected immediately after the intervention (at 60 days) from app participants. Patients entered the data on paper questionnaires, which was transferred into a purpose-built electronic database. Data management will be carried out using OpenClinica Enterprise v3.12.2.

As an incentive to complete and return the patient questionnaires, a £5 shopping voucher was sent in the post with each follow-up questionnaire, alongside a stamped addressed envelope. In the case that a questionnaire was not received, participants were sent a text message reminder. Nonresponders were then contacted by telephone in order to collect a subset of the questionnaire.

Participants allocated to the app groups were asked for permission to elaborate on the open comment boxes about app satisfaction, and discuss and prioritize clinical outcomes in two focus groups. The focus groups were held after the 6-month follow-up point finished. If participants were unable to attend a focus group, they were given the option to take part in a phone interview.

### Statistical Analyses

A full analysis plan can be found in Multimedia Appendix 3. Thirty participants were recruited to each of the three treatment groups, giving a total of 90 participants. As this is a feasibility study, we have not performed a sample size calculation based upon the power to detect a significant treatment effect on a clinical outcome. However, 90 participants should provide a reliable estimate for the standard deviation (SD) of the primary clinical outcome (likely to be pain acceptance) [43,44], which can be used to inform the sample size calculation of the main trial. Statistical analyses will be carried out using Stata 14.

**Table 1:** Meditation content over 60-day progressive course.

Series	Techniques involved
Take 10/Foundation 1 (first 10 days)	Open monitoring, body scan, breath as anchor
Foundation 2 (days 11-20)	As above, plus intention and altruism
Foundation 3 (days 21-30)	As above, plus integration of mindfulness with daily activities
Pain series (days, 31-60)	As above, plus visualization and enquiry (insight/Tibetan Vipassana)

#### Baseline

Baseline variables will be summarized for each treatment group using descriptive statistics.

#### Analysis of Feasibility Outcomes

The time taken to recruit all patients for the study and the number of participants recruited per month will be presented. An estimate of the SD of pain acceptance (CPAQ-8) in each treatment group at each follow up time point (60 days, 3 months, and 6 months) will be presented.

The proportion of patients in each treatment group who returned data at each follow-up time point (60 days, 3 months, and 6 months postrandomization) will be summarized. Summaries of baseline variables will be presented separately for patients who did and did not return data at each at the 6-month time point. Patient adherence outcomes and outcomes measuring usability of the app will be summarized separately for the intervention and active control treatment groups using descriptive statistics.

#### Analysis of Clinical Outcomes

For each clinical outcome we will present the number of patients in each treatment group with an observed outcome at each follow-up time point, and the mean (SD) in each treatment group at each follow-up time point. Estimates of treatment effect and 95% confidence intervals will be presented comparing the intervention group (mindfulness meditation app) to the control (treatment as usual) group, the intervention group to the active control (progressive muscle relaxation app) group, and the active control group to the control (treatment as usual) group.

Outcomes will be analyzed using linear mixed-effects models with outcome measurement (at three follow-up time points) as the dependent variable. The model will include fixed time effects, a fixed effect for treatment, time treatment interactions for 3-month and 6-month follow-up time points, and an unstructured correlation matrix for the residuals [45]. The model will include a baseline measure of the outcome as a covariate, assuming a linear relationship between baseline and outcome [46]. The model will be fitted using restricted maximum likelihood. Patient data will be analyzed according to the treatment group to which they were randomized (intention-to-treat). All patients with an observed outcome for at least one of the three follow-up time points (60 days, 3 months, or 6 months) will be included in the analysis [47]. If there are missing values for baseline measures of a clinical outcome, they will be replaced by the mean of the observed baseline values for all participants in all treatment arms (mean imputation) [48].

#### Qualitative Analysis

We will undertake a literal thematic analysis of the data from the focus group discussions to help us understand usability and implementation of, and response to, the intervention and research protocols [49,50] rather than developing or testing theory [51]. Features of app use and implementation issues will be summarized in a table of app features. For example, we will populate the row labelled *modular design* with comments on this specific feature. Columns of this table will represent more granular themes.

## Results

Recruitment for MEMPHIS took place between May 2016 and September 2016. The study was closed March 2017 and the report was submitted to the NIHR on October 26, 2017.

## Discussion

There are currently no rigorous RCTs that test mindfulness meditation interventions as a therapy for any chronic pain syndrome, including CPP. A previous systematic review [52] and our systematic review [11] identified no RCTs on mindfulness meditation in CPP. However, recent pilot studies [12,14,15] demonstrate promising outcomes and open the door for a large well-designed study with meaningful outcome measures. Given that psychological approaches, when combined with traditional therapies such as surgery, improve outcomes in CPP [7], mindfulness meditation is worthy of further investigation.

The intervention under investigation is novel in that it makes use of mindfulness meditation techniques as a treatment for CPP and it delivers this by means of a smartphone app (rather than traditional face-to-face therapy). Web- and smartphone-based health apps are a burgeoning field and offer the nonmedical population assistance in self-diagnosis, monitoring of long term medical conditions, or learning healthy behaviors. In the pain field alone, 279 smartphone apps were available for download in 2014, but these were simplistic, had unverified efficacy, and lacked the involvement of health care professionals in their development [53]. In our experience, no apps are currently incorporated into widespread routine clinical practice in CPP management. MEMPHIS presents a valuable opportunity to create a partnership between app development and health care professionals.

Not all patients with CPP would be expected to be highly confident in using smartphones and mobile technology, especially since women who are not usually using apps were recruited. Part of our data analysis, collected through focus groups, is directed towards the acceptability and usability of the app and will be valuable for any researcher planning future trials of smartphone technology in clinical interventions.

Given the ubiquity of the app, greater compliance with treatment and less wastage from patients not attending appointments may be expected. The use of the app in local primary, secondary, and tertiary care settings would be introduced in collaboration with general practitioner commissioning groups through local guidelines and protocols. Finally, if the app is shown to be effective in a full-scale trial, there would be benefit from studying how to extend the app to other pain conditions, such as headaches, back pain, and irritable bowel syndrome, in which face-to-face delivery of mindfulness meditation has had positive effects [9].

## Appendix

Multimedia Appendix 1 Amendments to protocol.

Multimedia Appendix 2 Case report forms to be used for collection of clinical outcome data.

Multimedia Appendix 3 Statistical analysis plan.

Multimedia Appendix 4 National Institute for Health Research peer review document.

## Abbreviations

CAMS-R: Cognitive and Mindfulness Scale - Revised
CPAQ-8: Chronic Pain Acceptance Questionnaire
CPP: chronic pelvic pain
HADS: Hospital Anxiety and Depression Scale
MBSR: mindfulness based stress reduction
MEMPHIS: Mindfulness Meditation for Women With Chronic Pelvic Pain
NHS: National Health Service
NIHR: National Institute for Health Research
PIS: Patient Information Sheet
PPI: patient and public involvement
RCT: randomized controlled trial
SD: standard deviation
SF-36: RAND 36-item Short Form health survey
SMD: standardized mean difference
SSM: supported self-management
SUS: System Usability Scale
