# Supplementary material for: Smartphone App Using Mindfulness Meditation for Women With Chronic Pelvic Pain (MEMPHIS): Protocol for a Randomized Feasibility Trial
Source: JMIR Res Protoc. 2018 Jan 15;7(1):e8. doi: 10.2196/resprot.7720 (PMC5789162; doi:10.2196/resprot.7720)
Supplement: Multimedia Appendix 1 [file resprot_v7i1e8_app1.pdf]

Changes to the protocol
£5 shopping vouchers were introduced as an incentive for participants to return follow up questionnaires.
Reminder texts were introduced to encourage a high return rate.
The collection of a smaller dataset over the phone was introduced for cases where women were unable to post questionnaires back.
An extension of a month was added to the trial timeline to allow enough time for analysis.
Quality of life scoring subscales used in the analysis were specified as Social functioning, Pain, General Health and Physical functioning using RAND SF-36.
The definition of '*app use*' for the adherence outcome '*Number of days of app use*' was changed from a session that is 50% completed to a session that is 90% completed. This was to correctly reflect the data that is collected from the app company.
Participants have been given a chance to answer questions over the phone if they are unable to attend focus groups.
