# Supplementary material for: Smartphone App Using Mindfulness Meditation for Women With Chronic Pelvic Pain (MEMPHIS): Protocol for a Randomized Feasibility Trial
Source: JMIR Res Protoc. 2018 Jan 15;7(1):e8. doi: 10.2196/resprot.7720 (PMC5789162; doi:10.2196/resprot.7720)
Supplement: Multimedia Appendix 2 [file resprot_v7i1e8_app2.pdf]

Participant ID No.: ___________

MEMPHIS

Feasibility study

BASELINE QUESTIONNAIRE

Confidential

Dear Participant,

The aim of this questionnaire is find out your current health state and get to know how you feel about living with chronic pain before you participate in the MEMPHIS study. You don't have to answer

any question you are not comfortable with but we would appreciate you completing as much as possible.

Please read the questions carefully. If you have any difficulties with the questionnaire please contact the study team.

Please write date completed: DD / MMM / YYYY

Participant ID No.: ___________

1. About you and your pain

Please tell us a bit about who you are:

**1.1 What is your age**	________Years
**1.2 What is your weight**	________ kg
**1.3 What is your height**	________ cm
**1.4 What are your living arrangements?** (please tick one box)	Alone ☐ With others ☐

**1.5 Which of the following best describes you?** (please tick one box)	Employed (full or part time, including self-employment)	☐	Unable to work due to long term sickness	☐
	Unemployed and looking for work	☐	Looking after your home/family	☐
	At school or in full time education	☐	Retired from paid work	☐
	Other (please specify)	☐	________________________	

**1.6 How old were you when you left full time education (e.g. school, college or university?** (please tick one box)	I did not receive a formal education	☐	Age 17 to 19	☐
	Age 12 or less	☐	Age 20 or over	☐
	Age 13 to 16	☐	I am still in full time education	☐
	Other (please specify)	☐	________________________	

**1.7. How long have you had your pain?** Please tick one box					
0-6 months	*7-12 months*	*1-2 years*	*3-5 years*	*6-10 years*	*More than 10 years*
☐	☐	☐	☐	☐	☐

**1.8. How would you rate your pain over the past week on a scale of 0 - 10, where 0 is 'No pain' and 10 is 'Pain as bad as could be'?** Please tick one box										
**No pain**										**Pain as bad as can be**
0	*1*	*2*	*3*	*4*	*5*	*6*	*7*	*8*	*9*	*10*
☐	☐	☐	☐	☐	☐	☐	☐	☐	☐	☐

Participant ID No.: ___________

2. Treatment of your pain

What types of treatments have you tried for your pain <u>in the last 6 months</u>?
(please indicate with a tick whether you have used the following:)

Treatment name	No	Yes	Treatment name	No	Yes
Acupuncture	☐	☐	Massage	☐	☐
Gabapentin	☐	☐	Nutrition/diet	☐	☐
Amitriptyline	☐	☐	Codeine or Morphine type painkillers	☐	☐
Biofeedback	☐	☐	Nerve blocks	☐	☐
Botox injection	☐	☐	Over the counter medication	☐	☐
Contraceptive pills/patch/ ring	☐	☐	Physiotherapy	☐	☐
Exercise, yoga or pilates	☐	☐	Psychological (talking) therapy	☐	☐
Injections to suppress ovaries (e.g. Prostap, Zoladex)	☐	☐	Transcutaneous Electrical Nerve Stimulation (TENS)	☐	☐
Herbal Medicine	☐	☐	Surgery	☐	☐
Meditation or relaxation exercises	☐	☐	Other – please state ________________________	☐	☐

Are you using any pain treatment currently? Yes ☐ No ☐

Participant ID No.: ___________

3. General Health

These questions ask for your views about your health. This information will help keep track of how you feel and how well you are able to do your usual activities.

Please answer every question by marking the answer as indicated. If you are unsure about how to answer a question, please give the best answer you can. When answering the question please asses how you felt in the last four weeks.

**3.1. In general, would you say your health is?** *(Please tick one box)*				
Excellent	***Very good***	***Good***	***Fair***	***Poor***
☐	☐	☐	☐	☐

**3.2. Compared to one year ago, how would you rate your health in general now?** *(Please tick one box)*				
Much better now than one year ago	***Somewhat better now than one year ago***	***About the same***	***Somewhat worse now than one year ago***	***Much worse than one year ago***
☐	☐	☐	☐	☐

3.3 The following items are about activities you might do during a typical day. Does your health now limit you in these activities? If so, how much?

**a. Vigorous activities, such as running, lifting heavy objects, participating in strenuous sports** *(Please tick one box)*		**b. Moderate activities, such as moving a table, pushing a vacuum cleaner, bowling, or playing golf** *(Please tick one box)*	
**Yes, limited a lot**	☐	**Yes, limited a lot**	☐
**Yes, limited a little**	☐	**Yes, limited a little**	☐
**No, not limited at all**	☐	**No, not limited at all**	☐
**c. Lifting or carrying groceries** *(Please tick one box)*		**d. Climbing several flights of stairs** *(Please tick one box)*	
**Yes, limited a lot**	☐	**Yes, limited a lot**	☐
**Yes, limited a little**	☐	**Yes, limited a little**	☐
**No, not limited at all**	☐	**No, not limited at all**	☐

Participant ID No.: ___________

General Health continued…

**e. Climbing one flight of stairs** *(Please tick one box)*		**f. Bending, kneeling or stooping** *(Please tick one box)*	
**Yes, limited a lot**	☐	**Yes, limited a lot**	☐
**Yes, limited a little**	☐	**Yes, limited a little**	☐
**No, not limited at all**	☐	**No, not limited at all**	☐
**g. Walking more than a mile** *(Please tick one box)*		**h. Walking several hundred yards** *(Please tick one box)*	
**Yes, limited a lot**	☐	**Yes, limited a lot**	☐
**Yes, limited a little**	☐	**Yes, limited a little**	☐
**No, not limited at all**	☐	**No, not limited at all**	☐
**i. Walking one hundred yards** *(Please tick one box)*		**j. Bathing or dressing yourself** *(Please tick one box)*	
**Yes, limited a lot**	☐	**Yes, limited a lot**	☐
**Yes, limited a little**	☐	**Yes, limited a little**	☐
**No, not limited at all**	☐	**No, not limited at all**	☐

3.4 During the **past 4 weeks,** have you had any of the following problems with your work or other regular daily activities as a result of your physical health?

**a. Cut down the amount of time you spent on work or other activities** *(Please tick one box)*		**b. Accomplished less than you would like** *(Please tick one box)*	
**All of the time**	☐	**All of the time**	☐
**Most of the time**	☐	**Most of the time**	☐
**Some of the time**	☐	**Some of the time**	☐
**A little of the time**	☐	**A little of the time**	☐
**None of the time**	☐	**None of the time**	☐

General Health continued…

**c. Were limited in the kind of work or other activities** *(Please tick one box)*		**d. Had difficulty performing the work or other activities (for example, it took extra effort)** *(Please tick one box)*	
**All of the time**	☐	**All of the time**	☐
**Most of the time**	☐	**Most of the time**	☐
**Some of the time**	☐	**Some of the time**	☐
**A little of the time**	☐	**A little of the time**	☐

Participant ID No.: ___________

None of the time ☐	None of the time ☐

3.5 During the **past 4 weeks**, have you had any of the following problems with your work or other regular daily activities as a result of any emotional problems (such as feeling depressed or anxious)?

**a. Cut down the amount of time you spent on work or other activities** *(Please tick one box)*		**b. Accomplished less than you would like** *(Please tick one box)*	
**All of the time**	☐	**All of the time**	☐
**Most of the time**	☐	**Most of the time**	☐
**Some of the time**	☐	**Some of the time**	☐
**A little of the time**	☐	**A little of the time**	☐
**None of the time**	☐	**None of the time**	☐

**c. Did work or other activities less carefully than usual** *(Please tick one box)*	
**All of the time**	☐
**Most of the time**	☐
**Some of the time**	☐
**A little of the time**	☐
**None of the time**	☐

Participant ID No.: ___________

General Health continued...

**3.6. During the past 4 weeks, to what extent has your physical health or emotional problems interfered with your normal social activities with family, friends, neighbours or groups?** *(Please tick one box)*				
Not at all	*Slightly*	*Moderately*	*Quite a bit*	*Extremely*
☐	☐	☐	☐	☐

**3.7. How much bodily pain have you had during the past 4 weeks?** *(Please tick one box)*					
None	*Very mild*	*Mild*	*Moderate*	*Severe*	*Very severe*
☐	☐	☐	☐	☐	☐

**3.8. During the past 4 weeks, how much did pain interfere with your normal work (including both work outside the home and housework)?** *(Please tick one box)*				
Not at all	*A little bit*	*Moderately*	*Quite a bit*	*Extremely*
☐	☐	☐	☐	☐

3.9. These questions are about how you feel and how things have been with you during **the past 4 weeks**. For each question, please give the answer that comes closest to the way you have been feeling.

**a. Did you feel full of life?** *(Please tick one box)*		**b. Have you been very nervous?** *(Please tick one box)*	
All of the time	☐	All of the time	☐
Most of the time	☐	Most of the time	☐
Some of the time	☐	Some of the time	☐
A little of the time	☐	A little of the time	☐
None of the time	☐	None of the time	☐
**c. Have you felt so down in the dumps that nothing could cheer you up?** *(Please tick one box)*		**d. Have you felt calm and peaceful?** *(Please tick one box)*	
All of the time	☐	All of the time	☐
Most of the time	☐	Most of the time	☐
Some of the time	☐	Some of the time	☐
A little of the time	☐	A little of the time	☐
None of the time	☐	None of the time	☐

General Health continued...

**e. Did you have lots of energy?** *(Please tick one box)*		**f. Have you felt downhearted and depressed?** *(Please tick one box)*	
All of the time	☐	All of the time	☐

Participant ID No.: ___________

Most of the time	☐	Most of the time	☐
Some of the time	☐	Some of the time	☐
A little of the time	☐	A little of the time	☐
None of the time	☐	None of the time	☐
**g. Did you feel worn out?** *(Please tick one box)*		**h. Have you been happy?** *(Please tick one box)*	
All of the time	☐	All of the time	☐
Most of the time	☐	Most of the time	☐
Some of the time	☐	Some of the time	☐
A little of the time	☐	A little of the time	☐
None of the time	☐	None of the time	☐
**i. Did you feel tired?** *(Please tick one box)*			
All of the time	☐		
Most of the time	☐		
Some of the time	☐		
A little of the time	☐		
None of the time	☐		

**3.10. During the past 4 weeks, how much of the time has your physical health or emotional problems interfered with your social activities (like visiting friends, relatives, etc.?)** *(Please tick one box)*				
All of the time	*Most of the time*	*Some of the time*	*A little of the time*	*None of the time*
☐	☐	☐	☐	☐

General Health continued…

3.11 How true or false is each of the following statements for you?

**a. I seem to get sick a little easier than other people** *(Please tick one box)*		**b. I am as healthy as anybody I know** *(Please tick one box)*	
Definitely true	☐	Definitely true	☐
Mostly true	☐	Mostly true	☐
Don't know	☐	Don't know	☐
Mostly false	☐	Mostly false	☐
Definitely false	☐	Definitely false	☐
**c. I expect my health to get worse** *(Please tick one box)*		**d. My health is excellent** *(Please tick one box)*	

Participant ID No.: ____________

**Definitely true**	☐	**Definitely true**	☐
**Mostly true**	☐	**Mostly true**	☐
**Don’t know**	☐	**Don’t know**	☐
**Mostly false**	☐	**Mostly false**	☐
**Definitely false**	☐	**Definitely false**	☐

Participant ID No.: ___________

4. Your main symptoms

4.1. Choose one or two symptoms (physical or mental), which bother you the most.

- Write them on the lines.

- Now consider how bad each symptom is, over the last week, and score it by ticking the number.

	0	1	2	3	4	5	6
**SYMPTOM 1** **(specify):** ________________	☐ As good as it could be	☐	☐	☐	☐	☐	☐ As bad as it could be
**SYMPTOM 2** **(specify):** ________________	☐ As good as it could be	☐	☐	☐	☐	☐	☐ As bad as it could be

4.2. Now choose one activity (physical, social or mental) that is important to you, and that your problem makes difficult or prevents you doing. Score how bad it has been in the last week.

	0	1	2	3	4	5	6
**ACTIVITY (specify):** ________________	☐ As good as it could be	☐	☐	☐	☐	☐	☐ As bad as it could be

4.3. Lastly how would you rate your general feeling of wellbeing during the last week?

	0	1	2	3	4	5	6
**FEELING OF WELLBEING**	☐ As good as it could be	☐	☐	☐	☐	☐	☐ As bad as it could be

4.4. How long have you had Symptom 1, either all the time or on and off? (*Please circle*)

0 - 4 weeks *5- 12 weeks* *13 weeks – 1 year* *Between 1 - 5years* *More than 5 years*

4.5. Are you taking any medication FOR THIS PROBLEM? (*Please circle*) **YES / NO**

IF YES

4.5. a. Please write in name of medication, and how much a day/week

Name: ______________________ *Dose:* ____________ *mg* ________ *day/week (please circle)*

Name: ______________________ *Dose:* ____________ *mg* ________ *day/week (please circle)*

Name: ______________________ *Dose:* ____________ *mg* ________ *day/week (please circle)*

Name: ______________________ *Dose:* ____________ *mg* ________ *day/week (please circle)*

4.5. b. Is cutting down this medication: (*Please circle*)

Not important	A bit important	Very important	Not applicable

IF **NO (to Q.4.5):**

4.5.c. Is avoiding medication for this problem: (*Please circle*)

Not important	a bit important	very important	not applicable

Participant ID No.: ___________

5. Mood

Please read each item and tick the reply which comes closest to how you have been feeling **<u>in the past week</u>**.

Don't take too long over your replies: your immediate response to each item will probably be more accurate than a long thought out response.

**5.1. I feel tense or "wound up"** *(Please tick <u>one</u> box)*		**5.2. I still enjoy the things I used to enjoy** *(Please tick <u>one</u> box)*	
**Most of the time**	☐	**Definitely as much**	☐
**A lot of the time**	☐	**Not quite as much**	☐
**From time to time, occasionally**	☐	**Only a little**	☐
**Not at all**	☐	**Hardly at all**	☐

**5.3. I get a sort of frightened feeling as if something awful is about to happen** *(Please tick <u>one</u> box)*		**5.4. I can laugh and see the funny side of things** *(Please tick <u>one</u> box)*	
**Very definitely and quite badly**	☐	**As much as I always could**	☐
**Yes, but not too badly**	☐	**Not quite so much now**	☐
**A little but it doesn't worry me**	☐	**Definitely not so much now**	☐
**Not at all**	☐	**Not at all**	☐

**5.5. Worrying thoughts go through my mind** *(Please tick <u>one</u> box)*		**5.6. I feel cheerful** *(Please tick <u>one</u> box)*	
**A great deal of the time**	☐	**Not at all**	☐
**A lot of the time**	☐	**Not often**	☐
**From time to time but not too often**	☐	**Sometimes**	☐
**Only occasionally**	☐	**Most of the time**	☐

Mood continued…

**5.7. I can sit at ease and feel relaxed** *(Please tick <u>one</u> box)*		**5.8. I feel as if I am slowed down** *(Please tick <u>one</u> box)*	
**Definitely**	☐	**Nearly all of the time**	☐
**Usually**	☐	**Very often**	☐

Not often	☐	Sometimes	☐
Not at all	☐	Not at all	☐

**5.9. I get a sort of frightened feeling like "butterflies" in the stomach** *(Please tick one box)*		**5.10. I have lost interest in my appearance** *(Please tick one box)*	
Not at all	☐	Definitely	☐
Occasionally	☐	I don't take as much care as I should	☐
Quite often	☐	I may not take quite as much care	☐
Very often	☐	I take just as much care as ever	☐

**5.11. I feel restless as though I have to be on the move** *(Please tick one box)*		**5.12. I look forward with enjoyment to things** *(Please tick one box)*	
Very much indeed	☐	As much as I ever did	☐
Quite a lot	☐	Rather less than I used to	☐
Not very much	☐	Definitely less than I used to	☐
Not at all	☐	Hardly at all	☐

**5.13. I get sudden feelings of panic** *(Please tick one box)*		**5.14. I can enjoy a good book or radio or TV programme** *(Please tick one box)*	
Very often indeed	☐	Often	☐
Quite often	☐	Sometimes	☐
Not very often	☐	Not often	☐
Not at all	☐	Very seldom	☐

Participant ID No.: ____________

6. Mental focus

Please respond to each item by marking one box per row.

Items		Rarely/ Not at All	Sometimes	Often	Almost Always
**6.1**	It is easy for me to concentrate on what I am doing.	☐	☐	☐	☐
**6.2**	I am preoccupied by the future.	☐	☐	☐	☐
**6.3**	I can tolerate emotional pain	☐	☐	☐	☐
**6.4**	I can accept things I cannot change.	☐	☐	☐	☐
**6.5**	I can usually describe how I feel at the moment in considerable detail.	☐	☐	☐	☐
**6.6**	I am easily distracted.	☐	☐	☐	☐
**6.7**	I am preoccupied by the past	☐	☐	☐	☐
**6.8**	It's easy for me to keep track of my thoughts and feelings.	☐	☐	☐	☐
**6.9**	I try to notice my thoughts without judging them	☐	☐	☐	☐
**6.10**	I am able to accept the thoughts and feelings I have.	☐	☐	☐	☐
**6.11**	I am able to focus on the present moment	☐	☐	☐	☐
**6.12**	I am able to pay close attention to one thing for a long period of time.	☐	☐	☐	☐

Participant ID No.: ___________

7. Pain acceptance

Below you will find a list of statements. Please rate the truth of each statement as it has applies to you by ticking one response, where 0 is 'never true' and 6 is 'always true'.

7.1. I am getting on with the business of living no matter what my level of pain is *(Please tick one box)*

Never true						**Always true**
0	*1*	*2*	*3*	*4*	*5*	*6*
☐	☐	☐	☐	☐	☐	☐

7.2. Although things have changed, I am living a normal life despite my chronic pain *(Please tick one box)*

Never true						**Always true**
0	*1*	*2*	*3*	*4*	*5*	*6*
☐	☐	☐	☐	☐	☐	☐

7.3. I lead a full life even though I have chronic pain *(Please tick one box)*

Never true						**Always true**
0	*1*	*2*	*3*	*4*	*5*	*6*
☐	☐	☐	☐	☐	☐	☐

7.4. Keeping my pain level under control takes first priority whenever I'm doing something *(Please tick one box)*

Never true						**Always true**
0	*1*	*2*	*3*	*4*	*5*	*6*
☐	☐	☐	☐	☐	☐	☐

7.5. Before I can make any serious plans, I have to get some control over my pain *(Please tick one box)*

Never true						**Always true**
0	*1*	*2*	*3*	*4*	*5*	*6*
☐	☐	☐	☐	☐	☐	☐

Pain acceptance (continued…)

7.6. When my pain increases, I can still take care of my responsibilities *(Please tick one box)*

Participant ID No.: ____________

Never true						Always true
0	*1*	*2*	*3*	*4*	*5*	*6*
☐	☐	☐	☐	☐	☐	☐

7.7. I avoid putting myself in situations where my pain might increase *(Please tick one box)*

Never true						Always true
0	*1*	*2*	*3*	*4*	*5*	*6*
☐	☐	☐	☐	☐	☐	☐

7.8. My worries and fears about what pain will do to me are true *(Please tick one box)*

Never true						Always true
0	*1*	*2*	*3*	*4*	*5*	*6*
☐	☐	☐	☐	☐	☐	☐

Participant ID No.: ___________

8. Pain in your daily life

8.1 In **the past six months,** how much has this pain interfered with your daily activities rated on a 1-10 scale where 0 is "No interference" and 10 is "unable to carry on activities"? *(Please tick* *one* *box)*

**No interference**										**Unable to carry on activities**
**0**	**1**	**2**	**3**	**4**	**5**	**6**	**7**	**8**	**9**	**10**
☐	☐	☐	☐	☐	☐	☐	☐	☐	☐	☐

8.2 In **the past six months**, how much has this pain changed your ability to take part in recreational, social and family activities where 0 is "No change" and 10 is “Extreme change”? *(Please tick* *one* *box)*

**No change**										**Extreme change**
**0**	**1**	**2**	**3**	**4**	**5**	**6**	**7**	**8**	**9**	**10**
☐	☐	☐	☐	☐	☐	☐	☐	☐	☐	☐

8.3 In **the past six months** how much has this pain changed your ability to work (including housework) where 0 is "No change" and 10 is "Extreme change"? *(Please tick* *one* *box)*

**No change**										**Extreme change**
**0**	**1**	**2**	**3**	**4**	**5**	**6**	**7**	**8**	**9**	**10**
☐	☐	☐	☐	☐	☐	☐	☐	☐	☐	☐

Participant ID No.: ___________

9. Confidence

Please indicate how **confident** you are that you can do the following things **at present**, despite the pain, where 0 is 'not at all confident' and 6 is 'completely confident'

** Remember, these questions are not asking whether or not you have been doing these things, but rather how confident you are that you could do them **at present**, despite the pain

9.1. I can enjoy things, despite the pain *(Please tick one box)*

Not confident						**completely confident**
0	*1*	*2*	*3*	*4*	*5*	*6*
☐	☐	☐	☐	☐	☐	☐

9.2. I can do most household chores (e.g. tidying up, washing dishes etc), despite the pain *(Please tick one box)*

Not confident						**completely confident**
0	*1*	*2*	*3*	*4*	*5*	*6*
☐	☐	☐	☐	☐	☐	☐

9.3. I can socialise with my friends or family members as often as I used to, despite the pain *(Please tick one box)*

Not confident						**completely confident**
0	*1*	*2*	*3*	*4*	*5*	*6*
☐	☐	☐	☐	☐	☐	☐

9.4. I can cope with my pain in most situations *(Please tick one box)*

Not confident						**completely confident**
0	*1*	*2*	*3*	*4*	*5*	*6*
☐	☐	☐	☐	☐	☐	☐

9.5. I can do some form of work, despite the pain ('work' includes housework, paid and unpaid work) *(Please tick one box)*

Not confident						**completely confident**
0	*1*	*2*	*3*	*4*	*5*	*6*
☐	☐	☐	☐	☐	☐	☐

Confidence (continued...)

9.6. I can still do many of the things I enjoy doing, such as hobbies or leisure activity, despite the pain *(Please tick one box)*

Participant ID No.: ____________

Not confident						completely confident
0	*1*	*2*	*3*	*4*	*5*	*6*
☐	☐	☐	☐	☐	☐	☐

9.7. I can cope with my pain without medication *(Please tick one box)*

Not confident						completely confident
0	*1*	*2*	*3*	*4*	*5*	*6*
☐	☐	☐	☐	☐	☐	☐

9.8. I can still accomplish most of my goals in life, despite the pain *(Please tick one box)*

Not confident						completely confident
0	*1*	*2*	*3*	*4*	*5*	*6*
☐	☐	☐	☐	☐	☐	☐

9.9. I can live a normal lifestyle, despite the pain *(Please tick one box)*

Not confident						completely confident
0	*1*	*2*	*3*	*4*	*5*	*6*
☐	☐	☐	☐	☐	☐	☐

9.10. I can gradually become more active despite the pain *(Please tick one box)*

Not confident						completely confident
0	*1*	*2*	*3*	*4*	*5*	*6*
☐	☐	☐	☐	☐	☐	☐

Participant ID No.: ___________

10. Intimate life

**Satisfaction**		
**10.1**	During <u>the past 4 weeks</u>, how satisfied were you with the frequency of your sexual activity (with or without a partner)?	☐ Very satisfied<br>☐ Somewhat satisfied<br>☐ Neither satisfied nor dissatisfied<br>☐ Somewhat dissatisfied<br>☐ Very dissatisfied
**10.2**	During <u>the past 4 weeks</u>, how satisfied in general have you been with your ability to have and enjoy sex with/without a partner?	☐ Very satisfied<br>☐ Somewhat satisfied<br>☐ Neither satisfied nor dissatisfied<br>☐ Somewhat dissatisfied<br>☐ Very dissatisfied<br>☐ I don't have a partner/<br>☐ I don't have sex without a partner
**Orgasm**		
**10.3**	During <u>the past 4 weeks</u>, when you had sexual activity, how much of the time did you experience orgasm?	☐ Never<br>☐ Rarely<br>☐ Sometimes<br>☐ Most of the time<br>☐ All of the time<br>☐ I did not have sexual activity
**10.4**	During <u>the past 4 weeks</u>, when you had sexual activity, how much of the time did you feel satisfied after sexual activity?	☐ Never<br>☐ Rarely<br>☐ Sometimes<br>☐ Most of the time<br>☐ All of the time<br>☐ I did not have sexual activity

Participant ID No.: ____________

Intimate life (continued...)

**Orgasm**		
**10.5**	During the past 4 weeks, when you experienced orgasm, how strong or intense was the orgasm on average?	☐ Did not experience any orgasms<br>☐ Mild<br>☐ Moderate<br>☐ Strong
**10.6**	During the past 4 weeks, how much of a problem was difficulty in having an orgasm?	☐ Not a problem<br>☐ Little of a problem<br>☐ Somewhat of a problem<br>☐ Very much of a problem<br>☐ I did not have sexual activity
**Desire**		
**10.7**	During the past 4 weeks, how much of a problem was lack of sexual interest?	☐ Not a problem<br>☐ Little of a problem<br>☐ Somewhat of a problem<br>☐ Very much of a problem<br>☐ I did not have sexual activity
**10.8**	During the past 4 weeks, how often did you desire sex (with or without a partner?)	☐ Never<br>☐ Once or twice<br>☐ 3-4 times<br>☐ 5-6 times<br>☐ More than 6 times
**10.9**	During the past 4 weeks, how much of a problem was inability to relax and enjoy sex?	☐ Not a problem<br>☐ Little of a problem<br>☐ Somewhat of a problem<br>☐ Very much of a problem<br>☐ I did not have sexual activity

Intimate life (continued...)

Participant ID No.: ____________

Pelvic problem interference

**10.10**	During the past 4 weeks, to what extent has your bleeding interfered with your normal or regular sexual activity (with or without a partner)?	☐ Not at all<br>☐ Slightly<br>☐ Moderately<br>☐ Quite a bit<br>☐ Extremely
**10.11**	During the past 4 weeks, to what extent has your pelvic pain or discomfort interfered with your normal or regular sexual activity (with or without a partner)?	☐ Not at all<br>☐ Slightly<br>☐ Moderately<br>☐ Quite a bit<br>☐ Extremely
**10.12**	During the past 4 weeks, to what extent have your pelvic problems overall interfered with your normal or regular sexual activity (with or without a partner)?	☐ Not at all<br>☐ Slightly<br>☐ Moderately<br>☐ Quite a bit<br>☐ Extremely

Participant ID No.: ___________

11. Additional details

*11.1. **Which group best describes your ethnic group?** (please tick <u>one</u> box only)*

**White**	☐	**Black**	☐
**Central Asian**	☐	**Middle Eastern**	☐
**Southern Asian**	☐	**Mixed**	☐
**Other ethnic group**	☐	**Do not wish to say**	☐

In order to understand better the mechanism you developed to cope with persistent pain, please answer below questions. The questionnaire is confidential and your answers will be used only for research purposes.

**11.2 Do you smoke?**	Yes ☐ No ☐
If *yes*, how many cigarettes per week? ________	
**11.3. Do you drink alcohol?**	Yes ☐ No ☐
If *yes*, how many units of alcohol do you drink per week? _________units	
Units Guide	*An average pint of beer/ cider (5%) = 3 units*<br>*A 250ml glass of wine (11%) = 3 units*<br>*A single measure (25ml) of spirits (e.g. vodka or gin) = 1 unit*

THANK YOU FOR FILLING IN THE QUESTIONNAIRE

Participant ID No.: ___________

FOLLOW-UP QUESTIONNAIRE

Confidential

Dear Participant,

The aim of this questionnaire is find out your current health state and get to know how you feel about living with chronic pain before you participate in the MEMPHIS study. You don't have to answer

any question you are not comfortable with but we would appreciate you completing as much as possible.

Please read the questions carefully. If you have any difficulties with the questionnaire please contact the study team.

Please write date completed: DD / MMM / YYYY

Participant ID No.: ___________

1. General Health

These questions ask for your views about your health. This information will help keep track of how you feel and how well you are able to do your usual activities.

Please answer <u>every question</u> by marking the answer as indicated. If you are unsure about how to answer a question, please give the best answer you can. When answering the question please asses how you felt <u>in the last four weeks.</u>

**1.1. In general, would you say your health is?** *(Please tick one box)*				
Excellent	*Very good*	*Good*	*Fair*	*Poor*
☐	☐	☐	☐	☐

**1.2. Compared to one year ago, how would you rate your health in general now?** *(Please tick one box)*				
Much better now than one year ago	*Somewhat better now than one year ago*	*About the same*	*Somewhat worse now than one year ago*	*Much worse than one year ago*
☐	☐	☐	☐	☐

1.3 The following items are about activities you might do during a typical day. Does your health now limit you in these activities? If so how much?

**a. Vigorous activities, such as running, lifting heavy objects, participating in strenuous sports** *(Please tick one box)*		**b. Moderate activities, such as moving a table, pushing a vacuum cleaner, bowling, or playing golf** *(Please tick one box)*	
**Yes, limited a lot**	☐	**Yes, limited a lot**	☐
**Yes, limited a little**	☐	**Yes, limited a little**	☐
**No, not limited at all**	☐	**No, not limited at all**	☐
**c. Lifting or carrying groceries** *(Please tick one box)*		**d. Climbing several flights of stairs** *(Please tick one box)*	
**Yes, limited a lot**	☐	**Yes, limited a lot**	☐
**Yes, limited a little**	☐	**Yes, limited a little**	☐
**No, not limited at all**	☐	**No, not limited at all**	☐

Participant ID No.: ___________

General Health continued…

**e. Climbing one flight of stairs** *(Please tick one box)*		**f. Bending, kneeling or stooping** *(Please tick one box)*	
**Yes, limited a lot**	☐	**Yes, limited a lot**	☐
**Yes, limited a little**	☐	**Yes, limited a little**	☐
**No, not limited at all**	☐	**No, not limited at all**	☐
**g. Walking more than a mile** *(Please tick one box)*		**h. Walking several hundred yards** *(Please tick one box)*	
**Yes, limited a lot**	☐	**Yes, limited a lot**	☐
**Yes, limited a little**	☐	**Yes, limited a little**	☐
**No, not limited at all**	☐	**No, not limited at all**	☐
**i. Walking one hundred yards** *(Please tick one box)*		**j. Bathing or dressing yourself** *(Please tick one box)*	
**Yes, limited a lot**	☐	**Yes, limited a lot**	☐
**Yes, limited a little**	☐	**Yes, limited a little**	☐
**No, not limited at all**	☐	**No, not limited at all**	☐

1.4 During the **past 4 weeks,** have you had any of the following problems with your work or other regular daily activities as a result of your physical health?

**a. Cut down the amount of time you spent on work or other activities** *(Please tick one box)*		**b. Accomplished less than you would like** *(Please tick one box)*	
**All of the time**	☐	**All of the time**	☐
**Most of the time**	☐	**Most of the time**	☐
**Some of the time**	☐	**Some of the time**	☐
**A little of the time**	☐	**A little of the time**	☐
**None of the time**	☐	**None of the time**	☐

General Health continued…

**c. Were limited in the kind of work or other activities** *(Please tick one box)*		**d. Had difficulty performing the work or other activities (for example, it took extra effort)** *(Please tick one box)*	
**All of the time**	☐	**All of the time**	☐
**Most of the time**	☐	**Most of the time**	☐
**Some of the time**	☐	**Some of the time**	☐
**A little of the time**	☐	**A little of the time**	☐

Participant ID No.: ___________

**None of the time** ☐	**None of the time** ☐

1.5 During the **past 4 weeks**, have you had any of the following problems with your work or other regular daily activities as a result of any emotional problems (such as feeling depressed or anxious)?

**a. Cut down the amount of time you spent on work or other activities** *(Please tick one box)*		**b. Accomplished less than you would like** *(Please tick one box)*	
**All of the time**	☐	**All of the time**	☐
**Most of the time**	☐	**Most of the time**	☐
**Some of the time**	☐	**Some of the time**	☐
**A little of the time**	☐	**A little of the time**	☐
**None of the time**	☐	**None of the time**	☐

**c. Did work or other activities less carefully than usual** *(Please tick one box)*	
**All of the time**	☐
**Most of the time**	☐
**Some of the time**	☐
**A little of the time**	☐
**None of the time**	☐

Participant ID No.: ___________

General Health continued…

**1.6. During the past 4 weeks, to what extent has your physical health or emotional problems interfered with your normal social activities with family, friends, neighbours or groups?** *(Please tick one box)*				
Not at all	*Slightly*	*Moderately*	*Quite a bit*	*Extremely*
☐	☐	☐	☐	☐

**1.7. How much bodily pain have you had during the past 4 weeks?** *(Please tick one box)*					
None	*Very mild*	*Mild*	*Moderate*	*Severe*	*Very severe*
☐	☐	☐	☐	☐	☐

**1.8. During the past 4 weeks, how much did pain interfere with your normal work (including both work outside the home and housework)?** *(Please tick one box)*				
Not at all	*A little bit*	*Moderately*	*Quite a bit*	*Extremely*
☐	☐	☐	☐	☐

1.9. These questions are about how you feel and how things have been with you during **the past 4 weeks**. For each question, please give the answer that comes closest to the way you have been feeling.

**a. Did you feel full of life?** *(Please tick one box)*		**b. Have you been very nervous?** *(Please tick one box)*	
All of the time	☐	All of the time	☐
Most of the time	☐	Most of the time	☐
Some of the time	☐	Some of the time	☐
A little of the time	☐	A little of the time	☐
None of the time	☐	None of the time	☐
**c. Have you felt so down in the dumps that nothing could cheer you up?** *(Please tick one box)*		**d. Have you felt calm and peaceful?** *(Please tick one box)*	
All of the time	☐	All of the time	☐
Most of the time	☐	Most of the time	☐
Some of the time	☐	Some of the time	☐
A little of the time	☐	A little of the time	☐
None of the time	☐	None of the time	☐

General Health continued…

**e. Did you have lots of energy?** *(Please tick one box)*	**f. Have you felt downhearted and depressed?** *(Please tick one box)*

Participant ID No.: ____________

All of the time ☐<br>Most of the time ☐<br>Some of the time ☐<br>A little of the time ☐<br>None of the time ☐	All of the time ☐<br>Most of the time ☐<br>Some of the time ☐<br>A little of the time ☐<br>None of the time ☐
**g. Did you feel worn out?** *(Please tick one box)*	**h. Have you been happy?** *(Please tick one box)*
All of the time ☐<br>Most of the time ☐<br>Some of the time ☐<br>A little of the time ☐<br>None of the time ☐	All of the time ☐<br>Most of the time ☐<br>Some of the time ☐<br>A little of the time ☐<br>None of the time ☐
**i. Did you feel tired?** *(Please tick one box)*	
All of the time ☐<br>Most of the time ☐<br>Some of the time ☐<br>A little of the time ☐<br>None of the time ☐	

1.10. During the past 4 weeks, how much of the time has your physical health or emotional problems interfered with your social activities (like visiting friends, relatives, etc.?) *(Please tick one box)*

All of the time	*Most of the time*	*Some of the time*	*A little of the time*	*None of the time*
☐	☐	☐	☐	☐

Participant ID No.: ___________

General Health continued…

1.11 How true or false is each of the following statements for you?

**a. I seem to get sick a little easier than other people** *(Please tick one box)*		**b. I am as healthy as anybody I know** *(Please tick one box)*	
**Definitely true**	☐	**Definitely true**	☐
**Mostly true**	☐	**Mostly true**	☐
**Don't know**	☐	**Don't know**	☐
**Mostly false**	☐	**Mostly false**	☐
**Definitely false**	☐	**Definitely false**	☐
**c. I expect my health to get worse** *(Please tick one box)*		**d. My health is excellent** *(Please tick one box)*	
**Definitely true**	☐	**Definitely true**	☐
**Mostly true**	☐	**Mostly true**	☐
**Don't know**	☐	**Don't know**	☐
**Mostly false**	☐	**Mostly false**	☐
**Definitely false**	☐	**Definitely false**	☐

Participant ID No.: ___________

2. Your main symptoms

2.1. Please circle the number to show how severe your problem has been IN THE LAST WEEK. This should be YOUR opinion, no-one else's! Please use the same symptoms and activity as on the baseline questionnaire.

**SYMPTOM 1**<br>**(specify):**<br>________________	**0**<br>☐<br>**As good as it could be**	**1**<br>☐	**2**<br>☐	**3**<br>☐	**4**<br>☐	**5**<br>☐	**6**<br>☐<br>**As bad as it could be**

**SYMPTOM 2**<br>**(specify):**<br>________________	**0**<br>☐<br>**As good as it could be**	**1**<br>☐	**2**<br>☐	**3**<br>☐	**4**<br>☐	**5**<br>☐	**6**<br>☐<br>**As bad as it could be**

**ACTIVITY (specify):**<br>________________	**0**<br>☐<br>**As good as it could be**	**1**<br>☐	**2**<br>☐	**3**<br>☐	**4**<br>☐	**5**<br>☐	**6**<br>☐<br>**As bad as it could be**

2.2. How would you rate your general feeling of wellbeing during the last week?

**FEELING OF WELLBEING**	**0**<br>☐<br>**As good as it could be**	**1**<br>☐	**2**<br>☐	**3**<br>☐	**4**<br>☐	**5**<br>☐	**6**<br>☐<br>**As bad as it could be**

2.3. If an important new symptom has appeared please describe it and mark how bad it is below.

**SYMPTOM 3**<br>**(specify):**<br>________________	**0**<br>☐<br>**As good as it could be**	**1**<br>☐	**2**<br>☐	**3**<br>☐	**4**<br>☐	**5**<br>☐	**6**<br>☐<br>**As bad as it could be**

The treatment you are receiving may not be the only thing affecting your problem. If there is anything else that you think is important, such as changes you have made yourself, or other things happening in your life, please write it here (write overleaf if you need more space):

2.4. Are you taking any medication FOR THIS PROBLEM? (*Please circle*) **YES / NO**

Participant ID No.: ___________

*IF **YES***

2.5. a. Please write in name of medication, and how much a day/week

Name: ______________________ Dose: ___________ mg ______ day/week (please circle)

Name: ______________________ Dose: ___________ mg ______ day/week (please circle)

Name: ______________________ Dose: ___________ mg ______ day/week (please circle)

Name: ______________________ Dose: ___________ mg ______ day/week (please circle)

Participant ID No.: ___________

3. Mood

Please read each item and tick the reply which comes closest to how you have been feeling **in the past week**.

Don't take too long over your replies: your immediate response to each item will probably be more accurate than a long thought out response.

**3.1. I feel tense or "wound up"** *(Please tick one box)*		**3.2. I still enjoy the things I used to enjoy** *(Please tick one box)*	
**Most of the time**	☐	**Definitely as much**	☐
**A lot of the time**	☐	**Not quite as much**	☐
**From time to time, occasionally**	☐	**Only a little**	☐
**Not at all**	☐	**Hardly at all**	☐

**3.3. I get a sort of frightened feeling as if something awful is about to happen** *(Please tick one box)*		**3.4. I can laugh and see the funny side of things** *(Please tick one box)*	
**Very definitely and quite badly**	☐	**As much as I always could**	☐
**Yes, but not too badly**	☐	**Not quite so much now**	☐
**A little but it doesn't worry me**	☐	**Definitely not so much now**	☐
**Not at all**	☐	**Not at all**	☐

**3.5. Worrying thoughts go through my mind** *(Please tick one box)*		**3.6. I feel cheerful** *(Please tick one box)*	
**A great deal of the time**	☐	**Not at all**	☐
**A lot of the time**	☐	**Not often**	☐
**From time to time but not too often**	☐	**Sometimes**	☐
**Only occasionally**	☐	**Most of the time**	☐

Participant ID No.: ___________

Mood continued…

**3.7. I can sit at ease and feel relaxed** *(Please tick one box)*		**3.8. I feel as if I am slowed down** *(Please tick one box)*	
**Definitely**	☐	**Nearly all of the time**	☐
**Usually**	☐	**Very often**	☐
**Not often**	☐	**Sometimes**	☐
**Not at all**	☐	**Not at all**	☐

**3.9. I get a sort of frightened feeling like "butterflies" in the stomach** *(Please tick one box)*		**3.10. I have lost interest in my appearance** *(Please tick one box)*	
**Not at all**	☐	**Definitely**	☐
**Occasionally**	☐	**I don't take as much care as I should**	☐
**Quite often**	☐	**I may not take quite as much care**	☐
**Very often**	☐	**I take just as much care as ever**	☐

**3.11. I feel restless as though I have to be on the move** *(Please tick one box)*		**3.12. I look forward with enjoyment to things** *(Please tick one box)*	
**Very much indeed**	☐	**As much as I ever did**	☐
**Quite a lot**	☐	**Rather less than I used to**	☐
**Not very much**	☐	**Definitely less than I used to**	☐
**Not at all**	☐	**Hardly at all**	☐

**3.13. I get sudden feelings of panic** *(Please tick one box)*		**3.14. I can enjoy a good book or radio or TV programme** *(Please tick one box)*	
**Very often indeed**	☐	**Often**	☐
**Quite often**	☐	**Sometimes**	☐
**Not very often**	☐	**Not often**	☐
**Not at all**	☐	**Very seldom**	☐

Participant ID No.: ____________

4. Mental focus

Please respond to each item by marking one box per row.

Items		Rarely/ Not at All	Sometimes	Often	Almost Always
**4.1**	It is easy for me to concentrate on what I am doing.	☐	☐	☐	☐
**4.2**	I am preoccupied by the future.	☐	☐	☐	☐
**4.3**	I can tolerate emotional pain	☐	☐	☐	☐
**4.4**	I can accept things I cannot change.	☐	☐	☐	☐
**4.5**	I can usually describe how I feel at the moment in considerable detail.	☐	☐	☐	☐
**4.6**	I am easily distracted.	☐	☐	☐	☐
**4.7**	I am preoccupied by the past	☐	☐	☐	☐
**4.8**	It's easy for me to keep track of my thoughts and feelings.	☐	☐	☐	☐
**4.9**	I try to notice my thoughts without judging them	☐	☐	☐	☐
**4.10**	I am able to accept the thoughts and feelings I have.	☐	☐	☐	☐
**4.11**	I am able to focus on the present moment	☐	☐	☐	☐
**4.12**	I am able to pay close attention to one thing for a long period of time.	☐	☐	☐	☐

Participant ID No.: ___________

5. Pain acceptance

Below you will find a list of statements. Please rate the truth of each statement as it has applies to you by ticking one response, where 0 is 'never true' and 6 is 'always true'.

5.1. I am getting on with the business of living no matter what my level of pain is *(Please tick one box)*

Never true						**Always true**
0	***1***	***2***	***3***	***4***	***5***	**6**
☐	☐	☐	☐	☐	☐	☐

5.2. Although things have changed, I am living a normal life despite my chronic pain *(Please tick one box)*

Never true						**Always true**
0	***1***	***2***	***3***	***4***	***5***	**6**
☐	☐	☐	☐	☐	☐	☐

5.3. I lead a full life even though I have chronic pain *(Please tick one box)*

Never true						**Always true**
0	***1***	***2***	***3***	***4***	***5***	**6**
☐	☐	☐	☐	☐	☐	☐

5.4. Keeping my pain level under control takes first priority whenever I'm doing something *(Please tick one box)*

Never true						**Always true**
0	***1***	***2***	***3***	***4***	***5***	**6**
☐	☐	☐	☐	☐	☐	☐

5.5. Before I can make any serious plans, I have to get some control over my pain *(Please tick one box)*

Never true						**Always true**
0	***1***	***2***	***3***	***4***	***5***	**6**
☐	☐	☐	☐	☐	☐	☐

Pain acceptance (continued…)

5.6. When my pain increases, I can still take care of my responsibilities *(Please tick one box)*

Participant ID No.: ____________

Never true						Always true
0	*1*	*2*	*3*	*4*	*5*	*6*
☐	☐	☐	☐	☐	☐	☐

5.7. I avoid putting myself in situations where my pain might increase *(Please tick one box)*

Never true						Always true
0	*1*	*2*	*3*	*4*	*5*	*6*
☐	☐	☐	☐	☐	☐	☐

5.8. My worries and fears about what pain will do to me are true *(Please tick one box)*

Never true						Always true
0	*1*	*2*	*3*	*4*	*5*	*6*
☐	☐	☐	☐	☐	☐	☐

Participant ID No.: ___________

6. Pain in your daily life

6.1 In **the past six months,** how much has this pain interfered with your daily activities rated on a 1-10 scale where 0 is "No interference" and 10 is "unable to carry on activities"? *(Please tick one box)*

**No interference**										**Unable to carry on activities**
**0**	**1**	**2**	**3**	**4**	**5**	**6**	**7**	**8**	**9**	**10**
☐	☐	☐	☐	☐	☐	☐	☐	☐	☐	☐

6.2 In **the past six months**, how much has this pain changed your ability to take part in recreational, social and family activities where 0 is "No change" and 10 is "Extreme change"? *(Please tick one box)*

**No change**										**Extreme change**
**0**	**1**	**2**	**3**	**4**	**5**	**6**	**7**	**8**	**9**	**10**
☐	☐	☐	☐	☐	☐	☐	☐	☐	☐	☐

6.3 In **the past six months** how much has this pain changed your ability to work (including housework) where 0 is "No change" and 10 is "Extreme change"? *(Please tick one box)*

**No change**										**Extreme change**
**0**	**1**	**2**	**3**	**4**	**5**	**6**	**7**	**8**	**9**	**10**
☐	☐	☐	☐	☐	☐	☐	☐	☐	☐	☐

Participant ID No.: ___________

7. Confidence

Please indicate how **confident** you are that you can do the following things **at present**, despite the pain, where 0 is 'not at all confident' and 6 is 'completely confident'

** Remember, these questions are not asking whether or not you have been doing these things, but rather how confident you are that you could do them **at present**, despite the pain

7.1. I can enjoy things, despite the pain *(Please tick one box)*

Not confident | **completely confident**

0	1	2	3	4	5	6
☐	☐	☐	☐	☐	☐	☐

7.2. I can do most household chores (e.g. tidying up, washing dishes etc), despite the pain *(Please tick one box)*

Not confident | **completely confident**

0	1	2	3	4	5	6
☐	☐	☐	☐	☐	☐	☐

7.3. I can socialise with my friends or family members as often as I used to, despite the pain *(Please tick one box)*

Not confident | **completely confident**

0	1	2	3	4	5	6
☐	☐	☐	☐	☐	☐	☐

7.4. I can cope with my pain in most situations *(Please tick one box)*

Not confident | **completely confident**

0	1	2	3	4	5	6
☐	☐	☐	☐	☐	☐	☐

7.5. I can do some form of work, despite the pain ('work' includes housework, paid and unpaid work) *(Please tick one box)*

Not confident | **completely confident**

0	1	2	3	4	5	6
☐	☐	☐	☐	☐	☐	☐

Confidence (continued…)

7.6. I can still do many of the things I enjoy doing, such as hobbies or leisure activity, despite the pain *(Please tick one box)*

Participant ID No.: ____________

Not confident						completely confident
0	*1*	*2*	*3*	*4*	*5*	*6*
☐	☐	☐	☐	☐	☐	☐

7.7. I can cope with my pain without medication *(Please tick one box)*

Not confident						completely confident
0	*1*	*2*	*3*	*4*	*5*	*6*
☐	☐	☐	☐	☐	☐	☐

7.8. I can still accomplish most of my goals in life, despite the pain *(Please tick one box)*

Not confident						completely confident
0	*1*	*2*	*3*	*4*	*5*	*6*
☐	☐	☐	☐	☐	☐	☐

7.9. I can live a normal lifestyle, despite the pain *(Please tick one box)*

Not confident						completely confident
0	*1*	*2*	*3*	*4*	*5*	*6*
☐	☐	☐	☐	☐	☐	☐

7.10. I can gradually become more active despite the pain *(Please tick one box)*

Not confident						completely confident
0	*1*	*2*	*3*	*4*	*5*	*6*
☐	☐	☐	☐	☐	☐	☐

Participant ID No.: ___________

8. Intimate life

**Satisfaction**	
**8.1** During <u>the past 4 weeks</u>, how satisfied were you with the frequency of your sexual activity (with or without a partner)?	☐ Very satisfied<br>☐ Somewhat satisfied<br>☐ Neither satisfied nor dissatisfied<br>☐ Somewhat dissatisfied<br>☐ Very dissatisfied
**8.2** During <u>the past 4 weeks</u>, how satisfied in general have you been with your ability to have and enjoy sex with/without a partner?	☐ Very satisfied<br>☐ Somewhat satisfied<br>☐ Neither satisfied nor dissatisfied<br>☐ Somewhat dissatisfied<br>☐ Very dissatisfied<br>☐ I don't have a partner/<br>☐ I don't have sex without a partner
**Orgasm**	
**8.3** During <u>the past 4 weeks</u>, when you had sexual activity, how much of the time did you experience orgasm?	☐ Never<br>☐ Rarely<br>☐ Sometimes<br>☐ Most of the time<br>☐ All of the time<br>☐ I did not have sexual activity
**8.4** During <u>the past 4 weeks</u>, when you had sexual activity, how much of the time did you feel satisfied after sexual activity?	☐ Never<br>☐ Rarely<br>☐ Sometimes<br>☐ Most of the time<br>☐ All of the time<br>☐ I did not have sexual activity

Intimate life (continued...)

Participant ID No.: ___________

**Orgasm**	
**8.5** During <u>the past 4 weeks,</u> when you experienced orgasm, how strong or intense was the orgasm on average?	☐ Did not experience any orgasms<br>☐ Mild<br>☐ Moderate<br>☐ Strong
**8.6** During <u>the past 4 weeks</u>, how much of a problem was difficulty in having an orgasm?	☐ Not a problem<br>☐ Little of a problem<br>☐ Somewhat of a problem<br>☐ Very much of a problem<br>☐ I did not have sexual activity

**Desire**	
**8.7** During <u>the past 4 weeks,</u> how much of a problem was lack of sexual interest?	☐ Not a problem<br>☐ Little of a problem<br>☐ Somewhat of a problem<br>☐ Very much of a problem<br>☐ I did not have sexual activity
**8.8** During <u>the past 4 weeks,</u> how often did you desire sex (with or without a partner?)	☐ Never<br>☐ Once or twice<br>☐ 3-4 times<br>☐ 5-6 times<br>☐ More than 6 times
**8.9** During <u>the past 4 weeks</u>, how much of a problem was inability to relax and enjoy sex?	☐ Not a problem<br>☐ Little of a problem<br>☐ Somewhat of a problem<br>☐ Very much of a problem<br>☐ I did not have sexual activity

Intimate life (continued…)

**Pelvic problem interference**

Participant ID No.: ____________

**8.10**	During <u>the past 4 weeks,</u> to what extent has your bleeding interfered with your normal or regular sexual activity (with or without a partner)?	☐ Not at all<br>☐ Slightly<br>☐ Moderately<br>☐ Quite a bit<br>☐ Extremely
**8.11**	During <u>the past 4 weeks,</u> to what extent has your pelvic pain or discomfort interfered with your normal or regular sexual activity (with or without a partner)?	☐ Not at all<br>☐ Slightly<br>☐ Moderately<br>☐ Quite a bit<br>☐ Extremely
**8.12**	During <u>the past 4 weeks,</u> to what extent have your pelvic problems overall interfered with your normal or regular sexual activity (with or without a partner)?	☐ Not at all<br>☐ Slightly<br>☐ Moderately<br>☐ Quite a bit<br>☐ Extremely

THANK YOU FOR FILLING IN THE QUESTIONNAIRE

MEMPHIS

Participant ID No.: ___________

APP USABILITY QUESTIONNAIRE

APP USABILITY QUESTIONNAIRE

Confidential

Dear Participant,

The following questionnaire will explore your current experience using the smartphone app. It will usually take 5 to 10 minutes to complete. It is comprised of ***two parts****:*

- ***Part 1:*** *consists of 10 brief questions with five response options*
- ***Part 2****: consists of 10 brief questions, with response options and comments sections*

We would appreciate you completing as much as possible.

Please read the questions carefully. If you have any difficulties with the questionnaire please contact the study team.

The questionnaire is confidential and your answers will be used only for research purposes.

Please write date completed: DD / MMM / YYYY

Participant ID No.: ___________

PART 1

System Usability Scale (SUS)

Participant ID No.: ________

1. *I think that I would like to use this app frequently (Please tick <u>one</u> box)*				
Totally disagree	*Somewhat disagree*	*Neither agree nor disagree*	*Somewhat agree*	*Totally agree*
1 ☐	2 ☐	3 ☐	4 ☐	5 ☐

2. *I found the app unnecessarily complex (Please tick <u>one</u> box)*				
Totally disagree	*Somewhat disagree*	*Neither agree nor disagree*	*Somewhat agree*	*Totally agree*
1 ☐	2 ☐	3 ☐	4 ☐	5 ☐

3. *I thought the app was easy to use (Please tick <u>one</u> box)*				
Totally disagree	*Somewhat disagree*	*Neither agree nor disagree*	*Somewhat agree*	*Totally agree*
1 ☐	2 ☐	3 ☐	4 ☐	5 ☐

4. *I think I would need the support of a technical person to be able to use this app (Please tick <u>one</u> box)*				
Totally disagree	*Somewhat disagree*	*Neither agree nor disagree*	*Somewhat agree*	*Totally agree*
1 ☐	2 ☐	3 ☐	4 ☐	5 ☐

5. *I found the various functions in the app worked well together (Please tick <u>one</u> box)*				
Totally disagree	*Somewhat disagree*	*Neither agree nor disagree*	*Somewhat agree*	*Totally agree*
1 ☐	2 ☐	3 ☐	4 ☐	5 ☐

6. *I thought there was too much inconsistency in this app (Please tick <u>one</u> box)*				
Totally disagree	*Somewhat disagree*	*Neither agree nor disagree*	*Somewhat agree*	*Totally agree*
1 ☐	2 ☐	3 ☐	4 ☐	5 ☐

7. *I would imagine that most people would learn to use this app very quickly (Please tick <u>one</u> box)*				
Totally disagree	*Somewhat disagree*	*Neither agree nor disagree*	*Somewhat agree*	*Totally agree*
1 ☐	2 ☐	3 ☐	4 ☐	5 ☐

8. *I found the app very cumbersome to use (Please tick <u>one</u> box)*				
Totally disagree	*Somewhat Disagree*	*Neither agree nor disagree*	*Somewhat agree*	*Totally agree*
1 ☐	2 ☐	3 ☐	4 ☐	5 ☐

9. *I felt very confident using the app (Please tick <u>one</u> box)*				
Totally disagree	*Somewhat Disagree*	*Neither agree nor disagree*	*Somewhat agree*	*Totally agree*

Participant ID No.: ___________

Final Score **(Only for research team)**

Participant ID No.: ___________

PART 2

App Satisfaction Measurement

1. It is easy to access the app whenever I wanted to use it (Please tick one box)				
Totally disagree	*Somewhat disagree*	*Neither agree nor disagree*	*Somewhat agree*	*Totally agree*
☐	☐	☐	☐	☐
2. After being shown, I understood how the app would work (Please tick one box)				
Totally disagree	*Somewhat disagree*	*Neither agree nor disagree*	*Somewhat agree*	*Totally agree*
☐	☐	☐	☐	☐
3. It was fun to work with the app (Please tick one box)				
Totally disagree	*Somewhat disagree*	*Neither agree nor disagree*	*Somewhat agree*	*Totally agree*
☐	☐	☐	☐	☐
4. The app worked well (Please tick one box)				
Totally disagree	*Somewhat disagree*	*Neither agree nor disagree*	*Somewhat agree*	*Totally agree*
☐	☐	☐	☐	☐
5. It was easy to work through the modules (Please tick one box)				
Totally disagree	*Somewhat disagree*	*Neither agree nor disagree*	*Somewhat agree*	*Totally agree*
☐	☐	☐	☐	☐
6. The number of modules was annoying (Please tick one box)				
Totally disagree	*Somewhat disagree*	*Neither agree nor disagree*	*Somewhat agree*	*Totally agree*
☐	☐	☐	☐	☐
7. The modules were well-displayed on my smartphone (Please tick one box)				

Participant ID No.: ____________

Totally disagree	*Somewhat disagree*	*Neither agree nor disagree*	*Somewhat agree*	*Totally agree*
☐	☐	☐	☐	☐

8. Using the app was difficult because of my daily activities (Please tick one box)

Totally disagree	*Somewhat disagree*	*Neither agree nor disagree*	*Somewhat agree*	*Totally agree*
☐	☐	☐	☐	☐

9. Using the app took too long (Please tick one box)

Totally disagree	*Somewhat disagree*	*Neither agree nor disagree*	*Somewhat agree*	*Totally agree*
☐	☐	☐	☐	☐

10. Did you use the app every day? If no, why: (Please tick one box)

Yes ☐

No ☐ If no, please say why:

THANK YOU FOR FILLING IN THE QUESTIONNAIRE

MEMPHIS

Feasibility study

Participant ID No.: ___________

6 MONTH FOLLOW-UP QUESTIONNAIRE

Confidential

Dear Participant,

The aim of this questionnaire is to find out your current health state and get to know how you feel about living with chronic pain before you participate in the MEMPHIS study. You don't have to answer

any question you are not comfortable with but we would appreciate you completing as much as possible.

Please read the questions carefully. If you have any difficulties with the questionnaire please contact the study team.

Please write date completed: DD / MMM / YYYY

Participant ID No.: ___________

9. General Health

These questions ask for your views about your health. This information will help keep track of how you feel and how well you are able to do your usual activities.

Please answer every question by marking the answer as indicated. If you are unsure about how to answer a question, please give the best answer you can. When answering the question please asses how you felt in the last four weeks.

**1.1. In general, would you say your health is?** *(Please tick one box)*				
Excellent	***Very good***	***Good***	***Fair***	***Poor***
☐	☐	☐	☐	☐

**1.2. Compared to one year ago, how would you rate your health in general now?** *(Please tick one box)*				
Much better now than one year ago	***Somewhat better now than one year ago***	***About the same***	***Somewhat worse now than one year ago***	***Much worse than one year ago***
☐	☐	☐	☐	☐

1.3 The following items are about activities you might do during a typical day. Does your health now limit you in these activities? If so how much?

**a. Vigorous activities, such as running, lifting heavy objects, participating in strenuous sports** *(Please tick one box)*		**b. Moderate activities, such as moving a table, pushing a vacuum cleaner, bowling, or playing golf** *(Please tick one box)*	
**Yes, limited a lot**	☐	**Yes, limited a lot**	☐
**Yes, limited a little**	☐	**Yes, limited a little**	☐
**No, not limited at all**	☐	**No, not limited at all**	☐
**c. Lifting or carrying groceries** *(Please tick one box)*		**d. Climbing several flights of stairs** *(Please tick one box)*	
**Yes, limited a lot**	☐	**Yes, limited a lot**	☐
**Yes, limited a little**	☐	**Yes, limited a little**	☐
**No, not limited at all**	☐	**No, not limited at all**	☐

Participant ID No.: ___________

General Health continued...

e. Climbing one flight of stairs *(Please tick one box)*		f. Bending, kneeling or stooping *(Please tick one box)*	
Yes, limited a lot	☐	Yes, limited a lot	☐
Yes, limited a little	☐	Yes, limited a little	☐
No, not limited at all	☐	No, not limited at all	☐
**g. Walking more than a mile** *(Please tick one box)*		**h. Walking several hundred yards** *(Please tick one box)*	
Yes, limited a lot	☐	Yes, limited a lot	☐
Yes, limited a little	☐	Yes, limited a little	☐
No, not limited at all	☐	No, not limited at all	☐
**i. Walking one hundred yards** *(Please tick one box)*		**j. Bathing or dressing yourself** *(Please tick one box)*	
Yes, limited a lot	☐	Yes, limited a lot	☐
Yes, limited a little	☐	Yes, limited a little	☐
No, not limited at all	☐	No, not limited at all	☐

1.4 During the **past 4 weeks,** have you had any of the following problems with your work or other regular daily activities as a result of your physical health?

a. Cut down the amount of time you spent on work or other activities *(Please tick one box)*		b. Accomplished less than you would like *(Please tick one box)*	
All of the time	☐	All of the time	☐
Most of the time	☐	Most of the time	☐
Some of the time	☐	Some of the time	☐
A little of the time	☐	A little of the time	☐
None of the time	☐	None of the time	☐

General Health continued...

c. Were limited in the kind of work or other activities *(Please tick one box)*		d. Had difficulty performing the work or other activities (for example, it took extra effort) *(Please tick one box)*	
All of the time	☐	All of the time	☐
Most of the time	☐	Most of the time	☐
Some of the time	☐	Some of the time	☐
A little of the time	☐	A little of the time	☐

Participant ID No.: ___________

**None of the time** ☐	**None of the time** ☐

1.5 During the **past 4 weeks**, have you had any of the following problems with your work or other regular daily activities as a result of any emotional problems (such as feeling depressed or anxious)?

**a. Cut down the amount of time you spent on work or other activities** *(Please tick one box)*		**b. Accomplished less than you would like** *(Please tick one box)*	
**All of the time**	☐	**All of the time**	☐
**Most of the time**	☐	**Most of the time**	☐
**Some of the time**	☐	**Some of the time**	☐
**A little of the time**	☐	**A little of the time**	☐
**None of the time**	☐	**None of the time**	☐

**c. Did work or other activities less carefully than usual** *(Please tick one box)*	
**All of the time**	☐
**Most of the time**	☐
**Some of the time**	☐
**A little of the time**	☐
**None of the time**	☐

Participant ID No.: ___________

General Health continued…

**1.6. During the <u>past 4 weeks</u>, to what extent has your physical health or emotional problems interfered with your normal social activities with family, friends, neighbours or groups?** *(Please tick <u>one</u> box)*				
Not at all	***Slightly***	***Moderately***	***Quite a bit***	***Extremely***
☐	☐	☐	☐	☐

**1.7. How much bodily pain have you had during the <u>past 4 weeks</u>?** *(Please tick <u>one</u> box)*					
None	***Very mild***	***Mild***	***Moderate***	***Severe***	***Very severe***
☐	☐	☐	☐	☐	☐

**1.8. During the <u>past 4 w</u>eeks, how much did pain interfere with your normal work (including both work outside the home and housework)?** *(Please tick <u>one</u> box)*				
Not at all	***A little bit***	***Moderately***	***Quite a bit***	***Extremely***
☐	☐	☐	☐	☐

1.9. These questions are about how you feel and how things have been with you during **<u>the past 4 weeks</u>**. For each question, please give the answer that comes closest to the way you have been feeling.

**a. Did you feel full of life?** *(Please tick <u>one</u> box)*		**b. Have you been very nervous?** *(Please tick <u>one</u> box)*	
All of the time	☐	All of the time	☐
Most of the time	☐	Most of the time	☐
Some of the time	☐	Some of the time	☐
A little of the time	☐	A little of the time	☐
None of the time	☐	None of the time	☐
**c. Have you felt so down in the dumps that nothing could cheer you up?** *(Please tick <u>one</u> box)*		**d. Have you felt calm and peaceful?** *(Please tick <u>one</u> box)*	
All of the time	☐	All of the time	☐
Most of the time	☐	Most of the time	☐
Some of the time	☐	Some of the time	☐
A little of the time	☐	A little of the time	☐
None of the time	☐	None of the time	☐

General Health continued…

**e. Did you have lots of energy?** *(Please tick <u>one</u> box)*	**f. Have you felt downhearted and depressed?** *(Please tick <u>one</u> box)*

Participant ID No.: ___________

All of the time	☐	All of the time	☐
Most of the time	☐	Most of the time	☐
Some of the time	☐	Some of the time	☐
A little of the time	☐	A little of the time	☐
None of the time	☐	None of the time	☐
**g. Did you feel worn out?** *(Please tick one box)*		**h. Have you been happy?** *(Please tick one box)*	
All of the time	☐	All of the time	☐
Most of the time	☐	Most of the time	☐
Some of the time	☐	Some of the time	☐
A little of the time	☐	A little of the time	☐
None of the time	☐	None of the time	☐
**i. Did you feel tired?** *(Please tick one box)*			
All of the time	☐		
Most of the time	☐		
Some of the time	☐		
A little of the time	☐		
None of the time	☐		

1.10. During the past 4 weeks, how much of the time has your physical health or emotional problems interfered with your social activities (like visiting friends, relatives, etc.?) *(Please tick one box)*

All of the time	*Most of the time*	*Some of the time*	*A little of the time*	*None of the time*
☐	☐	☐	☐	☐

Participant ID No.: ___________

General Health continued...

1.11 How true or false is each of the following statements for you?

**a. I seem to get sick a little easier than other people** *(Please tick one box)*		**b. I am as healthy as anybody I know** *(Please tick one box)*	
Definitely true	☐	Definitely true	☐
Mostly true	☐	Mostly true	☐
Don't know	☐	Don't know	☐
Mostly false	☐	Mostly false	☐
Definitely false	☐	Definitely false	☐
**c. I expect my health to get worse** *(Please tick one box)*		**d. My health is excellent** *(Please tick one box)*	
Definitely true	☐	Definitely true	☐
Mostly true	☐	Mostly true	☐
Don't know	☐	Don't know	☐
Mostly false	☐	Mostly false	☐
Definitely false	☐	Definitely false	☐

Participant ID No.: ___________

10. Your main symptoms

2.1. Please circle the number to show how severe your problem has been IN THE LAST WEEK. This should be YOUR opinion, no-one else's! Please use the same symptoms and activity as on the baseline questionnaire.

	0	1	2	3	4	5	6
**SYMPTOM 1 (specify):** ________________	☐ As good as it could be	☐	☐	☐	☐	☐	☐ As bad as it could be

	0	1	2	3	4	5	6
**SYMPTOM 2 (specify):** ________________	☐ As good as it could be	☐	☐	☐	☐	☐	☐ As bad as it could be

	0	1	2	3	4	5	6
**ACTIVITY (specify):** ________________	☐ As good as it could be	☐	☐	☐	☐	☐	☐ As bad as it could be

2.2. How would you rate your general feeling of wellbeing during the last week?

	0	1	2	3	4	5	6
**FEELING OF WELLBEING**	☐ As good as it could be	☐	☐	☐	☐	☐	☐ As bad as it could be

2.3. If an important new symptom has appeared please describe it and mark how bad it is below.

	0	1	2	3	4	5	6
**SYMPTOM 3 (specify):** ________________	☐ As good as it could be	☐	☐	☐	☐	☐	☐ As bad as it could be

The treatment you are receiving may not be the only thing affecting your problem. If there is anything else that you think is important, such as changes you have made yourself, or other things happening in your life, please write it here (write overleaf if you need more space):

2.4. Are you taking any medication FOR THIS PROBLEM? (*Please circle*) **YES / NO**

Participant ID No.: ___________

*IF **YES***

2.5. a. Please write in name of medication, and how much a day/week

Name: ______________________ *Dose: ___________ mg ______ day/week (please circle)*

Name: ______________________ *Dose: ___________ mg ______ day/week (please circle)*

Name: ______________________ *Dose: ___________ mg ______ day/week (please circle)*

Name: ______________________ *Dose: ___________ mg ______ day/week (please circle)*

Participant ID No.: ___________

11. Mood

Please read each item and tick the reply which comes closest to how you have been feeling **in the past week**.

Don't take too long over your replies: your immediate response to each item will probably be more accurate than a long thought out response.

**3.1. I feel tense or "wound up"** *(Please tick one box)*		**3.2. I still enjoy the things I used to enjoy** *(Please tick one box)*	
**Most of the time**	☐	**Definitely as much**	☐
**A lot of the time**	☐	**Not quite as much**	☐
**From time to time, occasionally**	☐	**Only a little**	☐
**Not at all**	☐	**Hardly at all**	☐

**3.3. I get a sort of frightened feeling as if something awful is about to happen** *(Please tick one box)*		**3.4. I can laugh and see the funny side of things** *(Please tick one box)*	
**Very definitely and quite badly**	☐	**As much as I always could**	☐
**Yes, but not too badly**	☐	**Not quite so much now**	☐
**A little but it doesn't worry me**	☐	**Definitely not so much now**	☐
**Not at all**	☐	**Not at all**	☐

**3.5. Worrying thoughts go through my mind** *(Please tick one box)*		**3.6. I feel cheerful** *(Please tick one box)*	
**A great deal of the time**	☐	**Not at all**	☐
**A lot of the time**	☐	**Not often**	☐
**From time to time but not too often**	☐	**Sometimes**	☐
**Only occasionally**	☐	**Most of the time**	☐

Participant ID No.: ___________

Mood continued…

**3.7. I can sit at ease and feel relaxed** *(Please tick <u>one</u> box)*		**3.8. I feel as if I am slowed down** *(Please tick <u>one</u> box)*	
Definitely	☐	Nearly all of the time	☐
Usually	☐	Very often	☐
Not often	☐	Sometimes	☐
Not at all	☐	Not at all	☐

**3.9. I get a sort of frightened feeling like "butterflies" in the stomach** *(Please tick <u>one</u> box)*		**3.10. I have lost interest in my appearance** *(Please tick <u>one</u> box)*	
Not at all	☐	Definitely	☐
Occasionally	☐	I don't take as much care as I should	☐
Quite often	☐	I may not take quite as much care	☐
Very often	☐	I take just as much care as ever	☐

**3.11. I feel restless as though I have to be on the move** *(Please tick <u>one</u> box)*		**3.12. I look forward with enjoyment to things** *(Please tick <u>one</u> box)*	
Very much indeed	☐	As much as I ever did	☐
Quite a lot	☐	Rather less than I used to	☐
Not very much	☐	Definitely less than I used to	☐
Not at all	☐	Hardly at all	☐

**3.13. I get sudden feelings of panic** *(Please tick <u>one</u> box)*		**3.14. I can enjoy a good book or radio or TV programme** *(Please tick <u>one</u> box)*	
Very often indeed	☐	Often	☐
Quite often	☐	Sometimes	☐
Not very often	☐	Not often	☐
Not at all	☐	Very seldom	☐

Participant ID No.: ____________

12. Mental focus

Please respond to each item by marking one box per row.

Items		Rarely/ Not at All	Sometimes	Often	Almost Always
**4.1**	It is easy for me to concentrate on what I am doing.	☐	☐	☐	☐
**4.2**	I am preoccupied by the future.	☐	☐	☐	☐
**4.3**	I can tolerate emotional pain	☐	☐	☐	☐
**4.4**	I can accept things I cannot change.	☐	☐	☐	☐
**4.5**	I can usually describe how I feel at the moment in considerable detail.	☐	☐	☐	☐
**4.6**	I am easily distracted.	☐	☐	☐	☐
**4.7**	I am preoccupied by the past	☐	☐	☐	☐
**4.8**	It's easy for me to keep track of my thoughts and feelings.	☐	☐	☐	☐
**4.9**	I try to notice my thoughts without judging them	☐	☐	☐	☐
**4.10**	I am able to accept the thoughts and feelings I have.	☐	☐	☐	☐
**4.11**	I am able to focus on the present moment	☐	☐	☐	☐
**4.12**	I am able to pay close attention to one thing for a long period of time.	☐	☐	☐	☐

Participant ID No.: ___________

13. Pain acceptance

Below you will find a list of statements. Please rate the truth of each statement as it has applies to you by ticking one response, where 0 is ‘never true’ and 6 is ‘always true’.

**5.1. I am getting on with the business of living no matter what my level of pain is** *(Please tick one box)*						
Never true						**Always true**
0	*1*	*2*	*3*	*4*	*5*	*6*
☐	☐	☐	☐	☐	☐	☐

**5.2. Although things have changed, I am living a normal life despite my chronic pain** *(Please tick one box)*						
Never true						**Always true**
0	*1*	*2*	*3*	*4*	*5*	*6*
☐	☐	☐	☐	☐	☐	☐

**5.3. I lead a full life even though I have chronic pain** *(Please tick one box)*						
Never true						**Always true**
0	*1*	*2*	*3*	*4*	*5*	*6*
☐	☐	☐	☐	☐	☐	☐

**5.4. Keeping my pain level under control takes first priority whenever I’m doing something** *(Please tick one box)*						
Never true						**Always true**
0	*1*	*2*	*3*	*4*	*5*	*6*
☐	☐	☐	☐	☐	☐	☐

**5.5. Before I can make any serious plans, I have to get some control over my pain** *(Please tick one box)*						
Never true						**Always true**
0	*1*	*2*	*3*	*4*	*5*	*6*
☐	☐	☐	☐	☐	☐	☐

Pain acceptance (continued…)

**5.6. When my pain increases, I can still take care of my responsibilities** *(Please tick one box)*

Participant ID No.: ___________

Never true						**Always true**
0	*1*	*2*	*3*	*4*	*5*	*6*
☐	☐	☐	☐	☐	☐	☐

5.7. I avoid putting myself in situations where my pain might increase *(Please tick one box)*

Never true						**Always true**
0	*1*	*2*	*3*	*4*	*5*	*6*
☐	☐	☐	☐	☐	☐	☐

5.8. My worries and fears about what pain will do to me are true *(Please tick one box)*

Never true						**Always true**
0	*1*	*2*	*3*	*4*	*5*	*6*
☐	☐	☐	☐	☐	☐	☐

Participant ID No.: ___________

14. Pain in your daily life

6.1 In **the past six months,** how much has this pain interfered with your daily activities rated on a 1-10 scale where 0 is "No interference" and 10 is "unable to carry on activities"? *(Please tick one box)*

**No interference**										**Unable to carry on activities**
**0**	**1**	**2**	**3**	**4**	**5**	**6**	**7**	**8**	**9**	**10**
☐	☐	☐	☐	☐	☐	☐	☐	☐	☐	☐

6.2 In **the past six months**, how much has this pain changed your ability to take part in recreational, social and family activities where 0 is "No change" and 10 is “Extreme change”? *(Please tick one box)*

**No change**										**Extreme change**
**0**	**1**	**2**	**3**	**4**	**5**	**6**	**7**	**8**	**9**	**10**
☐	☐	☐	☐	☐	☐	☐	☐	☐	☐	☐

6.3 In **the past six months** how much has this pain changed your ability to work (including housework) where 0 is "No change" and 10 is "Extreme change"? *(Please tick one box)*

**No change**										**Extreme change**
**0**	**1**	**2**	**3**	**4**	**5**	**6**	**7**	**8**	**9**	**10**
☐	☐	☐	☐	☐	☐	☐	☐	☐	☐	☐

Participant ID No.: ___________

15. Confidence

Please indicate how **<u>confident</u>** you are that you can do the following things **<u>at present</u>**, despite the pain, where 0 is 'not at all confident' and 6 is 'completely confident'

** Remember, these questions are not asking whether or not you have been doing these things, but rather how confident you are that you could do them **<u>at present</u>**, despite the pain

**7.1. I can enjoy things, despite the pain** *(Please tick <u>one</u> box)*						
Not confident						**completely confident**
0	*1*	*2*	*3*	*4*	*5*	*6*
☐	☐	☐	☐	☐	☐	☐

**7.2. I can do most household chores (e.g. tidying up, washing dishes etc), despite the pain** *(Please tick <u>one</u> box)*						
Not confident						**completely confident**
0	*1*	*2*	*3*	*4*	*5*	*6*
☐	☐	☐	☐	☐	☐	☐

**7.3. I can socialise with my friends or family members as often as I used to, despite the pain** *(Please tick <u>one</u> box)*						
Not confident						**completely confident**
0	*1*	*2*	*3*	*4*	*5*	*6*
☐	☐	☐	☐	☐	☐	☐

**7.4. I can cope with my pain in most situations** *(Please tick <u>one</u> box)*						
Not confident						**completely confident**
0	*1*	*2*	*3*	*4*	*5*	*6*
☐	☐	☐	☐	☐	☐	☐

**7.5. I can do some form of work, despite the pain ('work' includes housework, paid and unpaid work)** *(Please tick <u>one</u> box)*						
Not confident						**completely confident**
0	*1*	*2*	*3*	*4*	*5*	*6*
☐	☐	☐	☐	☐	☐	☐

Confidence (continued...)

**7.6. I can still do many of the things I enjoy doing, such as hobbies or leisure activity, despite the pain** *(Please tick <u>one</u> box)*

Participant ID No.: ___________

Not confident						completely confident
0	*1*	*2*	*3*	*4*	*5*	*6*
☐	☐	☐	☐	☐	☐	☐

7.7. I can cope with my pain without medication *(Please tick one box)*

Not confident						completely confident
0	*1*	*2*	*3*	*4*	*5*	*6*
☐	☐	☐	☐	☐	☐	☐

7.8. I can still accomplish most of my goals in life, despite the pain *(Please tick one box)*

Not confident						completely confident
0	*1*	*2*	*3*	*4*	*5*	*6*
☐	☐	☐	☐	☐	☐	☐

7.9. I can live a normal lifestyle, despite the pain *(Please tick one box)*

Not confident						completely confident
0	*1*	*2*	*3*	*4*	*5*	*6*
☐	☐	☐	☐	☐	☐	☐

7.10. I can gradually become more active despite the pain *(Please tick one box)*

Not confident						completely confident
0	*1*	*2*	*3*	*4*	*5*	*6*
☐	☐	☐	☐	☐	☐	☐

Participant ID No.: ___________

16. Intimate life

**Satisfaction**	
**8.1** During <u>the past 4 weeks</u>, how satisfied were you with the frequency of your sexual activity (with or without a partner)?	☐ Very satisfied<br>☐ Somewhat satisfied<br>☐ Neither satisfied nor dissatisfied<br>☐ Somewhat dissatisfied<br>☐ Very dissatisfied
**8.2** During <u>the past 4 weeks</u>, how satisfied in general have you been with your ability to have and enjoy sex with/without a partner?	☐ Very satisfied<br>☐ Somewhat satisfied<br>☐ Neither satisfied nor dissatisfied<br>☐ Somewhat dissatisfied<br>☐ Very dissatisfied<br>☐ I don't have a partner/<br>☐ I don't have sex without a partner
**Orgasm**	
**8.3** During <u>the past 4 weeks</u>, when you had sexual activity, how much of the time did you experience orgasm?	☐ Never<br>☐ Rarely<br>☐ Sometimes<br>☐ Most of the time<br>☐ All of the time<br>☐ I did not have sexual activity
**8.4** During <u>the past 4 weeks</u>, when you had sexual activity, how much of the time did you feel satisfied after sexual activity?	☐ Never<br>☐ Rarely<br>☐ Sometimes<br>☐ Most of the time<br>☐ All of the time<br>☐ I did not have sexual activity

Intimate life (continued...)

Participant ID No.: ___________

**Orgasm**		
**8.5**	During <u>the past 4 weeks,</u> when you experienced orgasm, how strong or intense was the orgasm on average?	☐ Did not experience any orgasms<br>☐ Mild<br>☐ Moderate<br>☐ Strong
**8.6**	During <u>the past 4 weeks</u>, how much of a problem was difficulty in having an orgasm?	☐ Not a problem<br>☐ Little of a problem<br>☐ Somewhat of a problem<br>☐ Very much of a problem<br>☐ I did not have sexual activity
**Desire**		
**8.7**	During <u>the past 4 weeks,</u> how much of a problem was lack of sexual interest?	☐ Not a problem<br>☐ Little of a problem<br>☐ Somewhat of a problem<br>☐ Very much of a problem<br>☐ I did not have sexual activity
**8.8**	During <u>the past 4 weeks,</u> how often did you desire sex (with or without a partner?)	☐ Never<br>☐ Once or twice<br>☐ 3-4 times<br>☐ 5-6 times<br>☐ More than 6 times
**8.9**	During <u>the past 4 weeks</u>, how much of a problem was inability to relax and enjoy sex?	☐ Not a problem<br>☐ Little of a problem<br>☐ Somewhat of a problem<br>☐ Very much of a problem<br>☐ I did not have sexual activity

Intimate life (continued…)

Pelvic problem interference

Participant ID No.: ____________

8.10 During the past 4 weeks, to what extent has your bleeding interfered with your normal or regular sexual activity (with or without a partner)?

- ☐ Not at all
- ☐ Slightly
- ☐ Moderately
- ☐ Quite a bit
- ☐ Extremely

8.11 During the past 4 weeks, to what extent has your pelvic pain or discomfort interfered with your normal or regular sexual activity (with or without a partner)?

- ☐ Not at all
- ☐ Slightly
- ☐ Moderately
- ☐ Quite a bit
- ☐ Extremely

8.12 During the past 4 weeks, to what extent have your pelvic problems overall interfered with your normal or regular sexual activity (with or without a partner)?

- ☐ Not at all
- ☐ Slightly
- ☐ Moderately
- ☐ Quite a bit
- ☐ Extremely

17. Smartphone app

**9.1. Did you use the smartphone app for MEMPHIS? *(Please tick one box)***	
Yes	***No***

☐	☐

9.2 If Yes, Do you think you received the new treatment app or the comparison treatment app?
(Please tick one box)

New treatment app	Comparison treatment app	Don't Know
☐	☐	☐

THANK YOU FOR FILLING IN THE QUESTIONNAIRE

MEMPHIS
