# Supplementary material for: Smartphone App Using Mindfulness Meditation for Women With Chronic Pelvic Pain (MEMPHIS): Protocol for a Randomized Feasibility Trial
Source: JMIR Res Protoc. 2018 Jan 15;7(1):e8. doi: 10.2196/resprot.7720 (PMC5789162; doi:10.2196/resprot.7720)
Supplement: Multimedia Appendix 4 [file resprot_v7i1e8_app4.pdf]

NHS
National Institute for Health Research

RfPB Competition 23

Peer Review Form

**Application Reference**	PB-PG-1013-32025
**Application Title**	Mindfulness meditation using a smart-phone application for women with chronic pelvic pain<br><br>MEMPHIS
**Applicant**	Miss Elizabeth Ball
**Applicant Organisation**	Barts Health NHS Trust
**Total Amount Requested**	£237,122.00
**Reviewer Number**	1

**Reviewer Expertise**	Researcher in the same/a very similar field<br>Clinician in the same/a very similar field<br>Methodologist

1. RELEVANCE of the proposed research

a) How relevant and important is the proposed research to the priorities and needs of the NHS, and does it offer a health/healthcare solution with demonstrable benefit to patients?
b) Does the application demonstrate an awareness and understanding of previous relevant research or developments in this area?
c) To what extent does the proposed research add distinct value or provide an advance to what is already known from other work currently in progress or already completed in this area?

This is a pilot study to assess feasibility of conducting a 3 arm randomised controlled trial to assess the effectiveness in mindfullness meditation delivered via a smartphone app for 60 days versus 2 control groups (comparison app with relaxing sounds but no medication and no app at all) in reducing or curing chronic pelvic pain and improving quality of life.

Chronic pelvic pain in women is a common clinical problem with often no demonstrable clinical cause and cure. Patients are also usually subjected to surgical interventions such as diagnostic laparoscopy and sometimes empirical hysterectomy with the associated cost and clinical risks from the treatment. Others are referred to Chronic Pelvic Pain clinics with long term input with expensive drug treatment to control their pain and/or psychological input. Therefore, a simple behavioural therapy with medication which can be delivered in an inexpensive manner using a smartphone app can potentially address some of the burden on the NHS if it can be proven to be cost effective. I therefore feel that this study deals with the priorities and needs of the NHS.

In relation to mindfullness meditation, this is a relatively new area of research for chronic pelvic pain. The applicants however are experienced and have previously undertaken research in the diagnosis and treatment of chronic pelvic pain. They have a sound track record in research in this field. Therefore, although the use of mindfullness meditation has not been explored extensively in the patients with chronic pelvic pain, it is still worthwhile to study the effectiveness of this intervention in such patients.

As stated above, little information on mindfullness meditation is known on women with chronic pelvic

pain and this is also highlighted in the systematic review conducted by the applicants to accompany this grant application, thereby justifiing the research.

---

2. QUALITY of the proposed work

2a. Research design

a) Is the proposed research of high quality and does it address the stated objectives?
b) How convincing and coherent is the proposed rationale and approach?
c) Is the proposed design and methodology for all elements of the research well defined, appropriate, valid, robust and feasible within the timeframe and resources requested?
d) What are the strengths and weaknesses of the research design as proposed?

The applicants have explicitly indicated that the present proposal is only a pilot study on whether it is possible to mount a larger definitive randomised trial on this topic. I think this is wise given that there is very little information on the effectiveness on this intervention on chronic pelvic pain at the present time. The method of recruitment from primary and tertiary care for women with chronic pelvic pain, randomisation, concealment of randomisation, selected study outcomes, data analysis are appropraite. Because women recruited into the mindfullness meditation arm are thought via a smartphone app, it is appropriate not to employ a cluster randomisation design. Prior data supplied by the applicant has shown that this method of meditation can be successfully taught via a smartphone app for other types of pain. The use of sham group will also differentiate any effect sizes observed to be due to the meditation intervention itself or to the use of the app. As this proposal is only to fund a pilot study, a sample size calculation is not required here. Indeed, one of the purpose of this pilot study is to obtain some background data (such as variances of the pain and quality of life scores) in order to calculate the sample size for the definitive randomised study later on.

Given that mindfullness meditation has been shown to have some beneficial effects for patients with back pain, headache, fibromyalgia and diabetic neuropathy, it is not unreasonable to investigate whether this relatively simple intervention can be used to manage patients with chronic pelvic pain. Therefore, I find the rationale for this proposal to be coherent and robust.

Despite the level of the funding requested, I think the level of resourses seeked is justifiable and the time frame proposed for the study is reasonable. Recruitment into studies for chronic pelvic pain is not always easy and I think the applicants are realistic with the time scale stated to recruit 90 subjects (30 subjects in each arm of the study) into this pilot study based on previous experiences of recruiting similar patients into other clinical trials. The only consideration that I would add is whether allowance should be made for any attrition from the study as patients with chronic pelvic pain are notoriously noted to drop out from such studies. This is especially so when the mindfullness meditation is delivered over a period of 60 days and the follow up extends to 6 months following this intervention.

Overall, the research design is robust but the only consideration I would advocate is the definition employed for women with chronic pelvic pain. For the purpose of this study, the applicants have defined women with chronic pelvic pain as those with such pain for more than 3 months in duration. Usually, such patients are defined as women with such symptom of at least 6 months in duration to ensure that the pain is genuinely chronic in nature so that women with transient pain are not investigated or treated unnecessarily. Some of the applicants are currently involved with the MEDAL (MRI to Establish Diagnosis Against Laparoscopy for chronic pelvic pain) study and the later definition of chronic pelvic pain is used for this study.

---

2. QUALITY of the proposed work

2b. Work plan and proposed management arrangements

a) Do the work plan and project management arrangements give confidence that proposed milestones will be met within the specified timeframe?
b) Can you identify any difficulties that the applicants are likely to encounter in meeting their milestones?
c) Have the major ethical, scientific, technical and organisational challenges, as well as any issues around intellectual property rights, been identified and will they be addressed adequately?
d) Are the necessary clinical, academic or organisational links needed to support the research, or help translate it into practice, in place?

I think the proposed milestones for the project are realistic and I am comfortable that the project management plans are sufficiently robust to ensure that these milestones can be met within the proposed time frame.

Formal ethical approval will need to be obtained for this study but I do not anticipate any difficulty with it as the proposed intervention is unlikely to be associated with any significant harm or adverse effects.

There also appears to be sufficient clinical and academic support given to this study from this proposal.

---

3. STRENGTH of the research team

a) Are the applicants familiar with the methodologies outlined in their application and are they well qualified to undertake the proposed work on the basis of track record in relevant areas (as judged by publication output and previous research funding)?
b) Does the research team have the necessary breadth and depth of expertise to deliver the planned work?
c) Are the roles of the team members clearly described, and is the overall team well coordinated?
d) If the lead applicant is inexperienced, does he/she have appropriate support (e.g. from their host institution and more senior colleagues) to deliver the work plan?

Most of the applicants listed in this proposal have a proven track record in delivering successfully conducted publically funded clinical studies (either for diagnostic studies or randomised controlled trials) in the area of chronic pelvic pain. They therefore have the breadth and depth of expertise in delivering such work.

The roles of the various team members listed have been clearly delineated and coordinated to ensure the maximum likelihood of a successful conduct of such a study. The use of the Chronic Pelvic Pain Patient group in the development of the protocol, participant information and recruitment documentation and pilot these amongst their groups to ensure that the study design meets their recommendations, in particular the questionnaires on health outcomes is a good idea to ensure that the important issues from the patient perspective are all adequately trialled and addressed at the pilot stage.

---

4. IMPACT of the proposed work

a) Have the applicants outlined an appropriate and adequate approach to disseminating the result of their research (including engaging with healthcare planners and/or policy makers)? Could this be developed further?
b) Considering the plans for dissemination, what is the likelihood of significant changes to general practice or the potential to contribute to future health gain for patients and the general population?
c) Have the applicants identified any new intellectual property that will be produced during the course of this research and are there plans to protect and exploit it?

The results from this pilot study will be sufficient to change clinical practice. The applicants have already indicated that these results will be used to inform the design the definitive randomised study if proven to be feasible. Therefore, the issue of dissemination of the study findings will be less relevant for this proposal although it does not mean that they cannot be reported in a peer reviewed biomedical journal.

The applicants have also identified the relevant potential interlectual property rights for both Headspace Meditation Ltd which is the company developing the smartphone app for the mindfullness meditation and Barts Health NHS trust generated from this study.

---

5. VALUE for money (justification for proposed costs)

a) Are the resources required for this research, including staffing, clearly justified? Are they essential for the work proposed?
b) Where the application offers arguments about financial benefits, please assess how realistic these might be.
c) Taking into account the expected benefits of the work proposed and the level of resources

requested, does this application represent good value for money?
d) If required, are funds requested for Support and Treatment Costs appropriate?

In my view, I think the resourses seeked for this study are justified and essential to allow delivery of the work plan within the time scale stated.

To accurately estimate the potential financial benefits from this intervention is difficult but it is likely that to be substanstial for the NHS if mindfullness meditation can be proven to be effective in the management of women with chronic pelvic pain.

Taking all aspects of cost and benefits into consideration, I am of the opinion that the proposed work and level of financial supported requested with reasonable value for money.

6. INVOLVEMENT of patients and the public

Where applicable (N.B. if there is no patient and public involvement, please address question d below)
a) What is your assessment of the patient and public involvement (if any) in the development of the application including involvement in: identifying the research topic; prioritising the research questions; preparing the application (e.g. contributing to the research design)?
b) What is your assessment of any proposed plans for patient and public involvement throughout the duration of the research? Can you identify particular strengths, weaknesses and/or areas for improvement?
c) Are the resources set aside for patient and public involvement appropriate - including plans for a training and support budget?
d) If there is no patient and public involvement in the application, what is your assessment of the reasons given for this?

The proposal has highlighted the use of the Chronic Pelvic Pain Patient group in the development of the protocol, participant information and recruitment documentation and pilot these amongst their groups to ensure that the study design meets their recommendations, in particular the questionnaires on health outcomes. This is a great idea to ensure that the important issues from the patient perspective are all adequately trialled and addressed at the pilot stage.

7. Additional comments

Do you have any other comments or suggestions for how the proposed research might be improved? If so, please indicate whether you see these as critical factors.

None

National Institute for Health Research

RfPB Competition 23

Peer Review Form

**Application Reference**	PB-PG-1013-32025
**Application Title**	Mindfulness meditation using a smart-phone application for women with chronic pelvic pain<br><br>MEMPHIS
**Applicant**	Miss Elizabeth Ball
**Applicant Organisation**	Barts Health NHS Trust
**Total Amount Requested**	£237,122.00
**Reviewer Number**	2

**Reviewer Expertise**	I am an academic psychologist, specialising in Health Psychology - health behaviour, physical activity, theories of health behaviour, chronic pain, disability.

1. RELEVANCE of the proposed research

a) How relevant and important is the proposed research to the priorities and needs of the NHS, and does it offer a health/healthcare solution with demonstrable benefit to patients?
b) Does the application demonstrate an awareness and understanding of previous relevant research or developments in this area?
c) To what extent does the proposed research add distinct value or provide an advance to what is already known from other work currently in progress or already completed in this area?

---

a) This is an interesting, timely and relevant proposal. Commentators such as Alan Kazdin (clinical psychologist and former president of the American Psychological Association) have noted how in many cases we have effective psychological interventions, but not sufficient capacity to deliver them to those who need them. Mindfulness meditation is rapidly developing an evidence base showing in many cases it can promote functioning and well-being in various clinical groups, but as a face-to-face psychological intervention it requires resources and trained clinicians. This feasibility study, if successful, could produce a tool which could be helpful to many who experience CPP.

However, I wish to point out (as a psychologist) that a psychological intervention CANNOT be shifted from a face-to-face to technological mode of delivery without losing some of its power – on p.17 the applicant states that face-to-face delivery of mindfulness training could be “replaced by cheaper and more flexible app-delivered training” – this is simply incorrect as technological delivery is an adjunct, not a replacement, for face-to-face work in psychological interventions. This can be seen in stepped-care IAPT services where online CBT has not replaced face-t0-face therapy, but is suggested for those with more mild conditions to save resources for face-to-face work with clients with more serious problems.

b) The application shows a good awareness of previous relevant work.

c) I believe that for this chronic condition, the study has the potential to add significant value to what is already known and would well-inform any future full-scale trial.

2. QUALITY of the proposed work

2a. Research design

a) Is the proposed research of high quality and does it address the stated objectives?
b) How convincing and coherent is the proposed rationale and approach?
c) Is the proposed design and methodology for all elements of the research well defined, appropriate, valid, robust and feasible within the timeframe and resources requested?
d) What are the strengths and weaknesses of the research design as proposed?

In my opinion the proposed study is well-designed scientifically for a feasibility study, and capable of meeting the objectives stated. The rationale is well-expressed and the research design proposed fits in well with it and the needs of the patient population, as well as the need to obtain certain information before deciding whether to mount a full-scale trial. The fact that it was informed by input from a patient group is a strength.

However, I have some concerns about the measures which I think the researchers must address. Firstly, the intervention is aiming to increase mindfulness, and so any effect it has on outcomes should occur because mindfulness has increased. However, there is no measurement of mindfulness in the entire study. There is a well-established measure of chronic pain acceptance (the CPAQ), but pain acceptance and mindfulness are different concepts. I would say it is essential to include a questionnaire measure of mindfulness in the assessment battery at each time point. Only then can it be established that the app actually did increase mindfulness, and that any effect on outcomes was mediated by mindfulness – otherwise we will not know whether the app actually achieves its aim or not in this population! There are a number of such psychometric scales available which provide a measure of mindfulness.

Secondly, I am not convinced that the proposed questionnaire measures for depression and anxiety are optimal. The Beck scales are well-regarded but were developed and normed to measure the severity of depression (BDI) and anxiety (BAI) in psychiatric populations who have presented for psychiatric treatment. However, CPP patients are not psychiatric patients and are not presenting because they are mentally ill. In addition, the BDI measures somatic symptoms of depression which may be experienced by people with a physical illness because of their illness and not depression (e.g. sleep disturbance, tiredness, etc.). This can create inflated and unreliable depression scores in those with physical illness (see Williams and Richardson, 1993, on inflated BDI scores in chronic pain). A better choice (and more widespread in the health psychology literature for these reasons) would be the Hospital Anxiety and Depression Scale (Zigmond and Snaith, 1983).

A further concern is that participants in the relaxation and no-app arms may be exposed to mindfulness anyway, due to its increasing prominence in society and the media, may already know about it through their own research into treatments for their pain, or may look into it themselves or be recommended to it by another practitioner or person (e.g. doctor, family member, etc.). It may be helpful to ask participants in these arms, at the end of the study, whether they attempted any meditation during the study. Otherwise the control conditions could be less reliable than hoped.

---

2. QUALITY of the proposed work

2b. Work plan and proposed management arrangements

a) Do the work plan and project management arrangements give confidence that proposed milestones will be met within the specified timeframe?
b) Can you identify any difficulties that the applicants are likely to encounter in meeting their milestones?
c) Have the major ethical, scientific, technical and organisational challenges, as well as any issues around intellectual property rights, been identified and will they be addressed adequately?
d) Are the necessary clinical, academic or organisational links needed to support the research, or help translate it into practice, in place?

As far as I can see the work plan and management arrangements seem sound and well-designed. There seem to be strong academic and healthcare links and the team seems to have access to good facilities. I do not have any concerns over this element of the proposal.

---

3. STRENGTH of the research team

a) Are the applicants familiar with the methodologies outlined in their application and are they well qualified to undertake the proposed work on the basis of track record in relevant areas (as judged by publication output and previous research funding)?
b) Does the research team have the necessary breadth and depth of expertise to deliver the planned work?
c) Are the roles of the team members clearly described, and is the overall team well coordinated?
d) If the lead applicant is inexperienced, does he/she have appropriate support (e.g. from their host institution and more senior colleagues) to deliver the work plan?

My main concern in this area is that this is a study rooted in the science of psychology, with the main concepts being psychological (i.e. mindfulness, pain acceptance, depression, anxiety, etc.). However, a glaring omission in the team is that there is no psychologist, and no-one with much specific expertise in mindfulness, other than the PI who according to the application is a consultant gynaecologist but has authored one systematic review. I think the lack of a psychologist on the research team is a very serious problem for this piece of research, and undermines confidence in the proposal.

There is a psychiatrist on the team but this is not a replacement for a psychologist (as psychiatry is restricted to the study of mental illness), and the information about Prof. Roehricht in the application does not suggest he has much expertise in mindfulness or the areas covered by the study – "body psychotherapy" is a psychodynamic approach to treating mental illness and other psychological problems, and is not related to mindfulness. In addition, chronic pain is not a mental illness – it involves psychology more than psychiatry.

The team desperately needs a psychologist added – it should be a health psychologist, clinical psychologist with expertise in mindfulness, or an academic psychologist with some knowledge of the area. It seems from their information in the application that the existing team members have limited experience with mindfulness. In addition, on p.15 it states that mindfulness is "a psychological therapy" but most psychologists involved in the area would not describe it as this; it would probably be described as a way of being that can be taught and developed through meditation (which may form part of an evidence-based therapeutic intervention, such as MBCT) to achieve beneficial effects on well-being and other outcomes. This and other elements I picked up indicate that understanding of mindfulness on the team may be good (for a medical doctor) but somewhat less specific than that of a psychologist. Issues such as those I picked up on in the "Quality" section also underline the need for a health psychologist or clinical psychologist as these could have been picked up prior to the application.

Apart from this the team looks strong. They are clearly experienced in research design and administration, in working with a CPP or chronic pain population, and in disseminating the results appropriately. This is in nearly every respect a fantastic team. However the lack of a psychologist is their Achilles' heel - this needs to be addressed. Once that is addressed my confidence in this team would be 100% (if not more!).

---

4. IMPACT of the proposed work

a) Have the applicants outlined an appropriate and adequate approach to disseminating the result of their research (including engaging with healthcare planners and/or policy makers)? Could this be developed further?
b) Considering the plans for dissemination, what is the likelihood of significant changes to general practice or the potential to contribute to future health gain for patients and the general population?
c) Have the applicants identified any new intellectual property that will be produced during the course of this research and are there plans to protect and exploit it?

The proposed dissemination plan makes sense, including the need to not be too forthright about the impact of mindfulness. Recent nationwide newspaper reports on the role of mindfulness in managing the impact of arthritis seem to underline this, because if patients become widely aware of the role of mindfulness it could make recruitment or controlled testing more difficult in a future RCT. While there is an ethical issue of withholding dissemination of an intervention that seems (in a preliminary study) to be promising, this is better addressed by the more scientifically rigorous RCT that may follow. Because the app is already developed, if found to be effective in the follow-on RCT it could be made available to patients very quickly and address issues of availability of treatment using this approach (though using it to wide access will not, as the authors erroneously state, replace face-to-face therapy). The researchers have considered the issue of intellectual property.

---

5. VALUE for money (justification for proposed costs)

a) Are the resources required for this research, including staffing, clearly justified? Are they essential for the work proposed?
b) Where the application offers arguments about financial benefits, please assess how realistic these might be.
c) Taking into account the expected benefits of the work proposed and the level of resources requested, does this application represent good value for money?
d) If required, are funds requested for Support and Treatment Costs appropriate?

This is an application for a fairly large grant, with most costs being staffing or institutional overheads (as usual in psychological studies, which this clearly bears a major relation to). I do not have experience with applying for large grants but I cannot see anything in this one which is unjustified or would make it poor value for money. App development and testing, even in commercial settings, is expensive, yet it is seen as an investment. My view is that given the potential scale of roll-out of such an app, this grant is a justifiable investment.

---

6. INVOLVEMENT of patients and the public

Where applicable (N.B. if there is no patient and public involvement, please address question d below)
a) What is your assessment of the patient and public involvement (if any) in the development of the application including involvement in: identifying the research topic; prioritising the research questions; preparing the application (e.g. contributing to the research design)?
b) What is your assessment of any proposed plans for patient and public involvement throughout the duration of the research? Can you identify particular strengths, weaknesses and/or areas for improvement?
c) Are the resources set aside for patient and public involvement appropriate - including plans for a training and support budget?
d) If there is no patient and public involvement in the application, what is your assessment of the reasons given for this?

I was pleased to see that CPP patients are involved at many stages of the research, and a pilot group provided information taken into account when planning the study. Offers to train patient representatives in necessary skills to fulfill their role (e.g. presentation skills) were generous and open-hearted and demonstrated something very positive to me when I read the application.

---

7. Additional comments

Do you have any other comments or suggestions for how the proposed research might be improved? If so, please indicate whether you see these as critical factors.

I would like to state that I see this as an exciting and timely proposal with the potential for a very positive impact on health - my comments above (primarily from a health psychology perspective) do not detract from my very positive overall impression of the proposal (though I think it is important that these points are addressed).

As a non-critical factor, unless it is usually paid by their university, allocating some grant funds for publication of a paper(s) in an open-access journal may be useful.

Finally, I would ask the researchers to consider on what smartphone platforms the app will be available. Not everyone who has such a device has an Apple iPhone or Android device (many apps are only available for these platforms). My own Windows Phone has a large and growing market share but fewer apps, as does Blackberry. Platform of device available to each participant, and which platforms the app works on, needs to be considered. For example, it may determine whether a person can be randomised to the app group (and if not, might this be a confounding variable – is there something different or unusual about Windows Phone/Blackberry owners??!).

National Institute for Health Research

RfPB Competition 23

Lay Review Form

**Application Reference**	PB-PG-1013-32025
**Application Title**	Mindfulness meditation using a smart-phone application for women with chronic pelvic pain<br><br>MEMPHIS
**Applicant**	Miss Elizabeth Ball
**Applicant Organisation**	Barts Health NHS Trust
**Total Amount Requested**	£237,122.00
**Reviewer Number**	3

**Reviewer Expertise**	Member of public with a more general view<br>Service user rep on NICE guidance development group on treatment of depression in people with chronic health problems.

1. RELEVANCE of the proposed research

a) How relevant and important is the proposed research to the priorities and needs of the NHS, and does it offer a health/healthcare solution with demonstrable benefit to patients?
b) Does the application demonstrate an awareness and understanding of previous relevant research or developments in this area?
c) To what extent does the proposed research add distinct value or provide an advance to what is already known from other work currently in progress or already completed in this area?

a) The use of the App may prove to be a healthcare benefit to patients with CPP with significant improvement to daily living and well-being. Furthermore it may result in reduction in costs when compared not only with high intensity interventions eg surgery and medication but also low intensity interventions eg acupuncture and TENS.

b) Applicants have emphasised that past studies have been mostly small. However they are aware of Mindful Meditation's beneficial assistance in other painful conditions (though delivered face to face). It is advantageous that they have already undertaken their own comprehensive systematic review. I would draw their attention to a paper entitled 'The cost of somatisation', F. Creed. (see my comment in 2 a) b).

c) The research may be valuable in that the App does not have negative side-effects unlike surgery and long term use of medication. A huge advantage is that it is so accessible ie on tap and does not entail lengthy appointments and going on waiting lists at for example pain management clinics.

2. QUALITY of the proposed work

2a. Research design

a) Is the proposed research of high quality and does it address the stated objectives?
b) How convincing and coherent is the proposed rationale and approach?
c) Is the proposed design and methodology for all elements of the research well defined, appropriate,

valid, robust and feasible within the timeframe and resources requested?
d) What are the strengths and weaknesses of the research design as proposed?

---

a) A feasibility rather than a pilot study would seem the right course of action. I believe for this condition, it is appropriate to cover primary, secondary and tertiary, patients presenting at or being referred to different settings. Plans for seeking consent, data collection and analysis are all sound.

b) The applicants have fully explained how CPP is difficult to treat and there are problems with capacity and waiting lists. It is a debillitating condition for patients having negative impacts on their lives and work. Somatisation has had some focus (I would draw the applicants' attention to a paper 'The cost of somatisation' Francis Creed which would make good background reading) and depression, stress and anxiety have also been discussed. Pain acceptance has been another focus. The flexibility of a Smartphone App may prove to be an important tool especially from a patient empowerment aspect. An innovative approach.

c) Conducting the MEMPHIS study to answer specific questions in preparation for the full scale study, would seem the best course of action. Well developed chart in Attachment 2 adds clarity to timescales envisaged and corresponds with text. I believe ample time has been allocated for analysis and writing up; meetings have been planned at appropriate intervals.

d) Strengths are the excellent ppi (see my comments under that section) and the relative simplicity of testing the feasibility of the App in clinical settings. A weakness would be the potential slow recruitment but in my view the applicants have demonstrated an awareness of this and have planned accordingly. Entry of data by patients seems risky. Again the applicants have pre-empted this by offering paper or facilitation by telephone. 90 participants is an appropriate number as sample size.

---

2. QUALITY of the proposed work

2b. Work plan and proposed management arrangements

a) Do the work plan and project management arrangements give confidence that proposed milestones will be met within the specified timeframe?
b) Can you identify any difficulties that the applicants are likely to encounter in meeting their milestones?
c) Have the major ethical, scientific, technical and organisational challenges, as well as any issues around intellectual property rights, been identified and will they be addressed adequately?
d) Are the necessary clinical, academic or organisational links needed to support the research, or help translate it into practice, in place?

---

a) The applicants have listed the key risks of reaching milestones and have acknowledged that issues such as ethics may delay the introduction of the MEMPHIS study and potentially the full scale trial. There is a commitment to ppi involvement in ethics which is commendable. Seeking ethics approval sooner rather than later is always preferable and the applicants have confirmed it will be prepared 6 months in advance of the study. Project management arrangements and responsibility thereof are in place.

b) There may be difficulties around adherence and drop-out but the applicants have shown an awareness of this. They have highlighted recruitment may be slower without the offer of the App. However they have confirmed that acceptability will be considered by the ppi.

c) The main disadvantage is that some patients will be allocated the sham app or no app at all. I would also question whether there are commissioning challenges. Could the packages be provided more cheaply in the future eg if Headspace were in competition.

d) The lay applicant has already made some important connections with a number of health organisations. Re NICE, I suggest establishing an open dialogue. NB I am unsure whether this would fall within the remit of technology or the Women & Children arm of NICE.

---

3. STRENGTH of the research team

a) Are the applicants familiar with the methodologies outlined in their application and are they well qualified to undertake the proposed work on the basis of track record in relevant areas (as judged by publication output and previous research funding)?
b) Does the research team have the necessary breadth and depth of expertise to deliver the planned

work?
c) Are the roles of the team members clearly described, and is the overall team well coordinated?
d) If the lead applicant is inexperienced, does he/she have appropriate support (e.g. from their host institution and more senior colleagues) to deliver the work plan?

---

a) Applicants are well qualified, have proven track records and published widely. The ppi member of the research team is at the core of the Pelvic Pain Support Network and is well known and respected in ppi circles.

b) I am pleased to see a trialist has been included on the research team. Clinical and academic aspects are both covered. I like the Abstract of Systematic Review (attachment 1) The lay applicant can bring an experiential expertise as well as an intellectual input.

c) I feel that patient safety should be covered in a more comprehensive way. The co-applicant responsible for ppi liaison could be allocated this resposibility as well as the lay co-applicant. This is important bearing in mind the number of patients who will not receive the intervention. Oversight (by trial manager of the study) including financial aspect have been covered.

d) The lead applicant has the necessary background and experience to conduct this research. A letter from Barts and the London School of Medicine confirming their support has been attached to the application.

---

4. IMPACT of the proposed work

a) Have the applicants outlined an appropriate and adequate approach to disseminating the result of their research (including engaging with healthcare planners and/or policy makers)? Could this be developed further?
b) Considering the plans for dissemination, what is the likelihood of significant changes to general practice or the potential to contribute to future health gain for patients and the general population?
c) Have the applicants identified any new intellectual property that will be produced during the course of this research and are there plans to protect and exploit it?

---

a) Excellant plans for dissemination to clinicians, commissioners and the public. A support group which could have an interest is The Womens Health Concern which is the patient arm of the British Psychological Society. An organisation called THOTH could be interested in such usage of technology.

b) It is envisaged that the lay co-applicant will be instrumental in dissemination through to implementation and practice. The strong contacts the reasearch team have already made with professional groups, stakeholders and the media, are reassuring.

c) New IP is the most important potential output of this study and co-applicants have already envisaged that it can be drawn in to updates on NHS pathways for patients in this group.

---

5. VALUE for money (justification for proposed costs)

a) Are the resources required for this research, including staffing, clearly justified? Are they essential for the work proposed?
b) Where the application offers arguments about financial benefits, please assess how realistic these might be.
c) Taking into account the expected benefits of the work proposed and the level of resources requested, does this application represent good value for money?
d) If required, are funds requested for Support and Treatment Costs appropriate?

---

a) Resources required are clear and salaries, increments, equipment and consummables, ppi (and arrangements for meetings) and estates costs have all been included. I believe all these components would be necessary (please note I do not have a financial background).

b) I believe the financial benefits are sound due to potential savings when compared with other interventions eg surgery and medication. Surrogate outcomes may relate to fewer appointments , fewer sickness absences and patients regaining the ability to work.

c) I was surprised at the research cost totalling almost a quarter of a million. However I do not have

any financial expertise so do not wish to comment further.

d) N/A
No funds requested under these two sections

---

6. INVOLVEMENT of patients and the public

Where applicable (N.B. if there is no patient and public involvement, please address question d below)
a) What is your assessment of the patient and public involvement (if any) in the development of the application including involvement in: identifying the research topic; prioritising the research questions; preparing the application (e.g. contributing to the research design)?
b) What is your assessment of any proposed plans for patient and public involvement throughout the duration of the research? Can you identify particular strengths, weaknesses and/or areas for improvement?
c) Are the resources set aside for patient and public involvement appropriate - including plans for a training and support budget?
d) If there is no patient and public involvement in the application, what is your assessment of the reasons given for this?

---

a) The lay co-applicant has, in my view, been pivotal in raising and developing awareness of the team, in work on clinical pathways for patients. I feel that the ppi in this application is particularly strong, having the necessary depth and breadth.

b) An experienced patient as a co-applicant is about as good as it gets. Presence of the lay co-applicant on the trial steering committee further strengthens the ppi. Any additional layering of ppi would have been too complex and hard to manage - they have pitched it just right, in my view. I love the concept of walking through the study (bottom of p12). My only criticism is that the role of the carer has not been fully debated (though dissemination to relatives has been mentioned). Carers may be a good source of support in the use of the App (and of course re the mechanics of technology, children). Carers are also important when it comes to decision making eg plans for travel (can be difficult for these patients) and also decisions on ability and return to work.

c) Good plans for training, remuneration and expenses (and in line with INVOLVE). Excellent plans for including ppi in dissemination. Support for presenting to a variety of audiences is often overlooked in research proposals but they have thought of this too. Direct and indirect costs have been highlighted under the finance section (no 14). However I do not feel qualified to comment on specifics.

d) N/A

---

7. Additional comments

Do you have any other comments or suggestions for how the proposed research might be improved? If so, please indicate whether you see these as critical factors.

I would have expected to see some minimal debate on other mechanisms eg TENS and also ergonomics. I also think some focus should have been placed on debate of problems around diagnostics (and false negatives / false positives) and situations where pelvic pain turns out to be unrelated to gynacological conditions.

I appreciate that there may be sensitive areas connected to this sort of research - pain experienced during intimacy, sexual health, pregnancy and fertility. But my impression is that the applicants are prepared. Is there an equality issue regarding excluding under 18s who do not have access to technology. Painful periods are a problem and medication does not always reach pain. However that is the only equality issue I have spotted.

NHS National Institute for Health Research

RfPB Competition 23

Peer Review Form

**Application Reference**	PB-PG-1013-32025
**Application Title**	Mindfulness meditation using a smart-phone application for women with chronic pelvic pain<br><br>MEMPHIS
**Applicant**	Miss Elizabeth Ball
**Applicant Organisation**	Barts Health NHS Trust
**Total Amount Requested**	£237,122.00
**Reviewer Number**	4

**Reviewer Expertise**	Clinician in the same/a very similar field<br>I am an anaesthetist with interest in Chronic pain. I believe in biopsychosocial approach to chronic pain management and selfcare.

1. RELEVANCE of the proposed research

a) How relevant and important is the proposed research to the priorities and needs of the NHS, and does it offer a health/healthcare solution with demonstrable benefit to patients?
b) Does the application demonstrate an awareness and understanding of previous relevant research or developments in this area?
c) To what extent does the proposed research add distinct value or provide an advance to what is already known from other work currently in progress or already completed in this area?

Research proposal is relevant to needs of NHS especially considering NHS needs to improve use of technology and self management. There is some evidence emerging that patient apps are useful in self management/ care but long term impact of their use is yet unknown. Mindfulness meditation has been shown to benefit chronic pain conditions, hence the concept is appropriate to be applied to CPP patients. It certainly adds value if indeed app based meditation learning has same impact as face to face teaching of meditation.

2. QUALITY of the proposed work

2a. Research design

a) Is the proposed research of high quality and does it address the stated objectives?
b) How convincing and coherent is the proposed rationale and approach?
c) Is the proposed design and methodology for all elements of the research well defined, appropriate, valid, robust and feasible within the timeframe and resources requested?
d) What are the strengths and weaknesses of the research design as proposed?

Proposed research certainly addresses the stated objectives. Design and methodology are well defined with resources and timeframe appropriate. It would be much more interesting study and probably more robust if comparison groups were face to face teaching of mindfulness meditation compared with use of apps. This would give better comparison of outcomes and better understanding of value (in the form of cost effectiveness) to NHS. Other issue is with techniques like

mindfulness meditation, patients need to use and get comfortable with these approaches for lot longer than 60 days to show any significant benefit in the outcome. It would be worthwhile thinking about using the app for longer than 60 days and then evaluate. Group set ups in face to face mediation also add benefits of peer support which are lost by using the apps. It is possible to work with Headspace Ltd. to explore the possibility of forming an online peer support network with consent of study participants.
The argument for study adding benefits is avoidance of face to face education and I am uncertain if this will be demonstrated by selected comparison groups.

---

2. QUALITY of the proposed work

2b. Work plan and proposed management arrangements

a) Do the work plan and project management arrangements give confidence that proposed milestones will be met within the specified timeframe?
b) Can you identify any difficulties that the applicants are likely to encounter in meeting their milestones?
c) Have the major ethical, scientific, technical and organisational challenges, as well as any issues around intellectual property rights, been identified and will they be addressed adequately?
d) Are the necessary clinical, academic or organisational links needed to support the research, or help translate it into practice, in place?

Yes, all the arrangements seem robust enough including support of people involved in the study. Challenge will be patient recruitment and adherence to use of the app without much support in the community support apart from reminders. This is a significant risk and researchers have identified this. I am uncertain how this will be mitigated with regards to timescales needed to recruit patients and throughput of this particular group of patients.

---

3. STRENGTH of the research team

a) Are the applicants familiar with the methodologies outlined in their application and are they well qualified to undertake the proposed work on the basis of track record in relevant areas (as judged by publication output and previous research funding)?
b) Does the research team have the necessary breadth and depth of expertise to deliver the planned work?
c) Are the roles of the team members clearly described, and is the overall team well coordinated?
d) If the lead applicant is inexperienced, does he/she have appropriate support (e.g. from their host institution and more senior colleagues) to deliver the work plan?

Research team appears to be strong and well supported with enough experience and expertise on board.

---

4. IMPACT of the proposed work

a) Have the applicants outlined an appropriate and adequate approach to disseminating the result of their research (including engaging with healthcare planners and/or policy makers)? Could this be developed further?
b) Considering the plans for dissemination, what is the likelihood of significant changes to general practice or the potential to contribute to future health gain for patients and the general population?
c) Have the applicants identified any new intellectual property that will be produced during the course of this research and are there plans to protect and exploit it?

Impact of this work in isolation will not be huge in terms of value to NHS. It also is unlikely to benefit patients in isolation without further support structures built around it. It certainly could be one of the measures with improved service design and other approaches to support self management of pain.

---

5. VALUE for money (justification for proposed costs)

a) Are the resources required for this research, including staffing, clearly justified? Are they essential for the work proposed?
b) Where the application offers arguments about financial benefits, please assess how realistic these might be.
c) Taking into account the expected benefits of the work proposed and the level of resources

requested, does this application represent good value for money?
d) If required, are funds requested for Support and Treatment Costs appropriate?

Resources requested are appropriate, yet considering value to NHS can not be demonstrated by comparison groups I significantly doubt it is good value for money.

---

6. INVOLVEMENT of patients and the public

Where applicable (N.B. if there is no patient and public involvement, please address question d below)
a) What is your assessment of the patient and public involvement (if any) in the development of the application including involvement in: identifying the research topic; prioritising the research questions; preparing the application (e.g. contributing to the research design)?
b) What is your assessment of any proposed plans for patient and public involvement throughout the duration of the research? Can you identify particular strengths, weaknesses and/or areas for improvement?
c) Are the resources set aside for patient and public involvement appropriate - including plans for a training and support budget?
d) If there is no patient and public involvement in the application, what is your assessment of the reasons given for this?

There is well anticipated patient and public involvement and a good plan for co-production of further research design. Resources for public involvement are appropriate too.

---

7. Additional comments

Do you have any other comments or suggestions for how the proposed research might be improved? If so, please indicate whether you see these as critical factors.

As explained above.

National Institute for Health Research

RfPB Competition 23

Peer Review Form

**Application Reference**	PB-PG-1013-32025
**Application Title**	Mindfulness meditation using a smart-phone application for women with chronic pelvic pain<br><br>MEMPHIS
**Applicant**	Miss Elizabeth Ball
**Applicant Organisation**	Barts Health NHS Trust
**Total Amount Requested**	£237,122.00
**Reviewer Number**	5

**Reviewer Expertise**	Researcher in the same/a very similar field<br>psychology, physical illness, online interventions, health services research, mental health

1. RELEVANCE of the proposed research

a) How relevant and important is the proposed research to the priorities and needs of the NHS, and does it offer a health/healthcare solution with demonstrable benefit to patients?
b) Does the application demonstrate an awareness and understanding of previous relevant research or developments in this area?
c) To what extent does the proposed research add distinct value or provide an advance to what is already known from other work currently in progress or already completed in this area?

a) The area of self-management with the aid of ehealth interventions is of great relevance to the priorities and needs of the NHS, and good quality research to support new initiatives is needed. However, I remain skeptical on whether the proposed research can contribute to the evidence base (as per my comments below).

b) The application fails to demonstrate awareness of mindfulness techniques, especially in a health app context. There is no evidence that the applicants chose the most appropriate app in comparison to others, there is also no evidence on the strength of the chosen app (e.g. what do the applicants know about the development process of the app or about the evidence base surrounding the app?).

2. QUALITY of the proposed work

2a. Research design

a) Is the proposed research of high quality and does it address the stated objectives?
b) How convincing and coherent is the proposed rationale and approach?
c) Is the proposed design and methodology for all elements of the research well defined, appropriate, valid, robust and feasible within the timeframe and resources requested?
d) What are the strengths and weaknesses of the research design as proposed?

An informed decision on the quality of the proposed work cannot be made as both the methodology and analysis plan lack clarity. I am not sure that the applicants have thought through all elements of

the study. By reading the research plan I am left with a lot of questions, just a couple of these are around the recruitment process and the intervention's (i.e. app's) modules.

2. QUALITY of the proposed work

2b. Work plan and proposed management arrangements

a) Do the work plan and project management arrangements give confidence that proposed milestones will be met within the specified timeframe?
b) Can you identify any difficulties that the applicants are likely to encounter in meeting their milestones?
c) Have the major ethical, scientific, technical and organisational challenges, as well as any issues around intellectual property rights, been identified and will they be addressed adequately?
d) Are the necessary clinical, academic or organisational links needed to support the research, or help translate it into practice, in place?

Once again, the questions above cannot be answered due to lack of detailed information.

---

3. STRENGTH of the research team

a) Are the applicants familiar with the methodologies outlined in their application and are they well qualified to undertake the proposed work on the basis of track record in relevant areas (as judged by publication output and previous research funding)?
b) Does the research team have the necessary breadth and depth of expertise to deliver the planned work?
c) Are the roles of the team members clearly described, and is the overall team well coordinated?
d) If the lead applicant is inexperienced, does he/she have appropriate support (e.g. from their host institution and more senior colleagues) to deliver the work plan?

---

a) It seems that the applicants have trial management experience, however I remain uncertain on whether they are able to undertake the proposed work. Please see further comments below.

b) Most of the applicants show general experience in 'trial and data management', with some others showing general 'clinical and academic expertise'. I am not convinced that the applicants have the necessary expertise or support required for such a project. In particular I am surprised that the team has no input from psychologists, considering their claim that mindfulness is a psychological approach. In addition, such a project requires considerable knowledge about ehealth, which is not evidenced by any of the applicants.

c) No, there is no clear description of each applicant's role in the suggested project.

d) The lead applicant lacks experience for such a project. As her main expertise and input is in gynaecology it would have been advisable to fulfil remaining expertise needs by selecting a team appropriate for all elements of the research suggested.

---

4. IMPACT of the proposed work

a) Have the applicants outlined an appropriate and adequate approach to disseminating the result of their research (including engaging with healthcare planners and/or policy makers)? Could this be developed further?
b) Considering the plans for dissemination, what is the likelihood of significant changes to general practice or the potential to contribute to future health gain for patients and the general population?
c) Have the applicants identified any new intellectual property that will be produced during the course of this research and are there plans to protect and exploit it?

Completely dependent on the results!

---

5. VALUE for money (justification for proposed costs)

a) Are the resources required for this research, including staffing, clearly justified? Are they essential for the work proposed?
b) Where the application offers arguments about financial benefits, please assess how realistic these might be.

c) Taking into account the expected benefits of the work proposed and the level of resources requested, does this application represent good value for money?
d) If required, are funds requested for Support and Treatment Costs appropriate?

Based on the proposal’s lack of clarity I would say poor value for money.

---

6. INVOLVEMENT of patients and the public

Where applicable (N.B. if there is no patient and public involvement, please address question d below)
a) What is your assessment of the patient and public involvement (if any) in the development of the application including involvement in: identifying the research topic; prioritising the research questions; preparing the application (e.g. contributing to the research design)?
b) What is your assessment of any proposed plans for patient and public involvement throughout the duration of the research? Can you identify particular strengths, weaknesses and/or areas for improvement?
c) Are the resources set aside for patient and public involvement appropriate - including plans for a training and support budget?
d) If there is no patient and public involvement in the application, what is your assessment of the reasons given for this?

Adequate PPI.

---

7. Additional comments

Do you have any other comments or suggestions for how the proposed research might be improved? If so, please indicate whether you see these as critical factors.

I would suggest that future applications by the applicants are carefully checked for typos.
